# Oral Misoprostol Versus Vaginal Dinoprostone for Labor Induction: A Systematic Review and Meta‐Analysis

**DOI:** 10.1111/jog.70369

**Published:** 2026-07-24

**Authors:** Tiffany Yeretsian, Hussain Mogharbel, Rayyan Rozzah, Rizwana Ashraf, Abirami Kirubarajan, Kristian Thorlund, Rohan D'Souza

**Affiliations:** ^1^ Department of Obstetrics & Gynecology McMaster University Hamilton Ontario Canada; ^2^ Division of Maternal‐Fetal Medicine, Department of Obstetrics & Gynaecology, Mount Sinai Hospital University of Toronto Toronto Ontario Canada; ^3^ Department of Obstetrics & Gynaecology King Abdulaziz University Jeddah Saudi Arabia; ^4^ Division of Gynecologic Oncology, Department of Obstetrics & Gynaecology McGill University Health Center Montreal Canada; ^5^ Department of Health Research Methods, Evidence and Impact McMaster University Hamilton Canada

**Keywords:** cervical ripening, labor induction, oral misoprostol, vaginal dinoprostone

## Abstract

**Aim:**

Pharmacologic cervical ripening is frequently used for labor induction, yet the comparative effectiveness, safety, and resource utilization of available agents remain uncertain. This review compared oral misoprostol with vaginal dinoprostone for labor induction in singleton pregnancies at ≥ 34 weeks' gestation.

**Methods:**

We conducted a systematic review and meta‐analysis of randomized controlled trials, searching 11 databases to May 2025. Eligible studies compared oral misoprostol with vaginal dinoprostone in singleton pregnancies ≥ 34 weeks undergoing induction without contraindications to vaginal birth. Seven critical outcomes were assessed: cesarean birth, uterine hyperstimulation, 5‐min Apgar score < 7, neonatal intensive care unit (NICU) admission, oxytocin augmentation, vaginal birth within 24 h, and induction‐to‐birth interval. Study selection and data extraction were performed in duplicate. Random‐effects meta‐analysis was applied, with risk of bias, certainty of evidence, and subgroup credibility assessed using Cochrane RoB 2.0, GRADE, and ICEMAN.

**Results:**

Eleven trials including 3783 participants were analyzed. Oral misoprostol was associated with lower cesarean birth (RR 0.83, 95% CI 0.74–0.94) and reduced oxytocin augmentation (RR 0.89, 95% CI 0.82–0.97). Vaginal dinoprostone was associated with higher vaginal birth within 24 h (RR 0.91, 95% CI 0.86–0.96) and a shorter induction‐to‐birth interval. No significant differences were observed in uterine hyperstimulation, 5‐min Apgar score < 7, or NICU admission.

**Conclusion:**

Oral misoprostol is associated with reduced cesarean birth and probably reduces the need for oxytocin augmentation, while vaginal dinoprostone is associated with higher rates of vaginal birth within 24 h and probably a shorter induction‐to‐birth interval. Maternal and neonatal safety outcomes appear comparable; these findings support consideration of oral misoprostol in selected populations and settings, while acknowledging underlying clinical and methodological heterogeneity.

## 1 Introduction

Labor induction is a common obstetric intervention, performed in approximately one in four pregnancies in high‐income countries [1, 2]. In low‐ and middle‐income countries, overall induction rates tend to be lower, but rates may be substantially higher in urban or tertiary referral centers [1, 2, 3]. Induction is indicated when the risks of continuing pregnancy outweigh the benefits of expectant management, necessitating timely intervention. In individuals with an unfavorable cervix, pharmacologic agents are commonly employed to promote cervical ripening and initiate uterine contractions.

Prostaglandins are widely recommended for cervical ripening. Vaginal dinoprostone, a synthetic prostaglandin E2 (PGE2) analogue, is commonly used but presents logistical challenges, including the need for refrigeration, higher cost, and vaginal or intracervical administration. These factors may limit its feasibility in resource‐constrained settings [4].

Misoprostol, a prostaglandin E1 (PGE1) analogue originally developed for gastric ulcer prevention, has gained popularity for labor induction due to its low cost, thermostability, and multiple routes of administration, including oral, buccal, vaginal, and rectal options [5]. These pharmacologic advantages enhance its utility in low‐resource environments. However, the optimal dosing regimen, safety profile, and comparative efficacy of oral misoprostol remain subjects of ongoing investigation [6, 7, 8, 9].

Previous systematic reviews have compared misoprostol, dinoprostone, and mechanical methods such as Foley or Cook balloon catheters; however, many pooled different routes of misoprostol administration or focused on vaginal misoprostol, limiting route‐specific conclusions [9, 10, 11, 12, 13, 14, 15]. Some reviews reported similar safety profiles for vaginal misoprostol and dinoprostone, although misoprostol was associated with higher rates of uterine tachysystole [9, 12]. Others found that oral misoprostol was associated with fewer neonatal adverse outcomes or concluded that oral misoprostol and vaginal dinoprostone demonstrated comparable safety and efficacy. However, these reviews often lacked formal assessments of risk of bias, certainty of evidence, or credibility of subgroup effects [14, 15].

This systematic review and meta‐analysis addresses these limitations by directly comparing oral misoprostol and vaginal dinoprostone for labor induction in singleton pregnancies at ≥ 34 weeks' gestation. Seven patient‐important outcomes were prioritized: cesarean birth, uterine hyperstimulation, 5‐min Apgar score < 7, neonatal intensive care unit (NICU) admission, oxytocin augmentation, vaginal delivery within 24 h, and induction‐to‐birth interval. The analysis incorporates rigorous methodology, including duplicate independent review, formal risk of bias assessment [16], certainty of evidence grading [17], and appraisal of subgroup effect credibility [18]. This review aims to provide a clinically relevant evidence synthesis to inform practice and policy, particularly in settings where cost, storage requirements, and administration logistics are critical considerations.

## 2 Methods

This systematic review and meta‐analysis was conducted in accordance with the PRISMA 2020 (Preferred Reporting Items for Systematic Reviews and Meta‐Analyses) guidelines (Appendix S35) and the Cochrane Handbook for Systematic Reviews of Interventions [19, 20]. The protocol was prospectively registered with PROSPERO (CRD42020168483).

### 2.1 Eligibility Criteria

Randomized controlled trials (RCTs) comparing oral misoprostol and vaginal dinoprostone for labor induction in individuals with singleton pregnancies at ≥ 34 weeks' gestation were eligible for inclusion. Studies were included regardless of dose, dosing frequency, or formulation of the intervention to ensure comprehensive capture of clinically relevant regimens. Trials involving participants with prior cesarean birth or uterine surgery were excluded, as were nonrandomized, quasi‐randomized, or cluster‐randomized designs. No restrictions were placed on language, publication year, or geographic setting.

### 2.2 Search Strategy

A comprehensive search was conducted across 11 databases: MEDLINE, Embase, Scopus, PubMed‐in‐Process, Web of Science, CENTRAL, Cochrane Database of Systematic Reviews, CINAHL, Emcare, Google Scholar, and ClinicalTrials.gov. The search strategy, developed in consultation with a medical information specialist, combined controlled vocabulary and keyword terms related to labor induction, misoprostol, and dinoprostone. Gray literature was searched using the CADTH Gray Matters checklist [21] and the YODA Project [22]. The final search was completed on May 27, 2025. Full search strategies are provided in the supplemental material (Appendices S1–S11).

### 2.3 Study Selection and Data Extraction

Two reviewers (T.Y., R.A.) independently screened titles and abstracts, followed by full‐text review of potentially eligible studies. Data extraction was performed independently and in duplicate using a standardized form. Extracted data included study characteristics, participant demographics, intervention details, and all reported outcomes. Risk of bias was similarly assessed independently by the two reviewers (T.Y., R.A.) using the Cochrane Risk of Bias 2.0 tool [16]. Discrepancies were resolved through discussion with a third reviewer (R.D.).

### 2.4 Outcomes

Seven critical outcomes were identified a priori as essential for decision‐making: cesarean birth (primary effectiveness outcome), uterine hyperstimulation (primary safety outcome), 5‐min Apgar score < 7 (safety outcome), NICU admission (safety outcome), oxytocin augmentation (primary resource‐related outcome), vaginal birth within 24 h (resource‐related outcome), and induction‐to‐vaginal birth interval (resource‐related outcome).

Additional important outcomes included vaginal birth within 48 h, instrumental birth, postpartum hemorrhage, non‐reassuring fetal heart tracings, need for tocolysis, maternal side effects (e.g., nausea, vomiting, diarrhea, shivering, fever), 1‐min Apgar score < 7, uterine tachysystole, uterine hypertonus, meconium‐stained amniotic fluid, and analgesia use.

The critical and important outcomes were defined a priori using standardized obstetric definitions where possible. Where studies reported differing definitions, outcomes were harmonized to standardized definitions when possible. Detailed definitions are provided in Appendix S12.

### 2.5 Data Synthesis and Statistical Analysis

Meta‐analyses were conducted using random‐effects models to account for between‐study heterogeneity. Risk ratios (RRs) were calculated for binary outcomes and mean differences (MDs) for continuous outcomes, each with 95% confidence intervals (CIs). Means and standard deviations were estimated from medians and interquartile ranges using validated methods.

Heterogeneity was assessed both statistically and clinically. Statistical heterogeneity was assessed using Cochran's *Q* test (*p* < 0.10) and quantified using the *I* ^2^ statistic, with thresholds of approximately 25%, 50%, and 75% representing low, moderate, and high heterogeneity, respectively [23]. Clinical heterogeneity was also assessed, including variation in dosing regimens, participant characteristics (e.g., parity), and study setting (e.g., country income level). These prespecified factors informed subgroup and sensitivity analyses. Both statistical (*I* ^2^ values) and clinical sources of heterogeneity were explicitly considered when interpreting results.

All analyses were conducted using DataParty software, with a pre‐determined significance threshold of 0.05 for the *p*‐value of the pooled effect estimate [24]. The certainty of evidence for each outcome was evaluated using the Core GRADE (Grading of Recommendations, Assessment, Development and Evaluation) framework, considering risk of bias, inconsistency, indirectness, imprecision, and publication bias [17]. Summary of Findings tables were generated using GRADEpro [25].

### 2.6 Subgroup Analyses and ICEMAN Assessment

Subgroup analyses were prespecified and conducted on an exploratory basis to investigate potential sources of heterogeneity, including parity (nulliparous versus multiparous) and country income level (high‐income versus low‐ and middle‐income countries). These analyses were not intended to provide confirmatory evidence of effect modification, and results were interpreted cautiously.

The Instrument for Assessing the Credibility of Effect Modification Analyses (ICEMAN) tool was used to examine the quality of the subgroup effects reported [18]. Each subgroup effect was assigned a credibility rating ranging from “Very Low” to “High” based on the responses to the questions in the ICEMAN checklist. Two reviewers (T.Y., R.A.) independently applied the ICEMAN tool to each subgroup analysis, with discrepancies resolved by consensus.

### 2.7 Sensitivity Analysis

Sensitivity analyses were performed by excluding studies at high risk of bias to assess the robustness of the findings. These analyses were conducted for all critical and important outcomes, in accordance with the Cochrane Handbook for Systematic Reviews of Interventions [26].

## 3 Results

### 3.1 Study Selection

**FIGURE 1:**
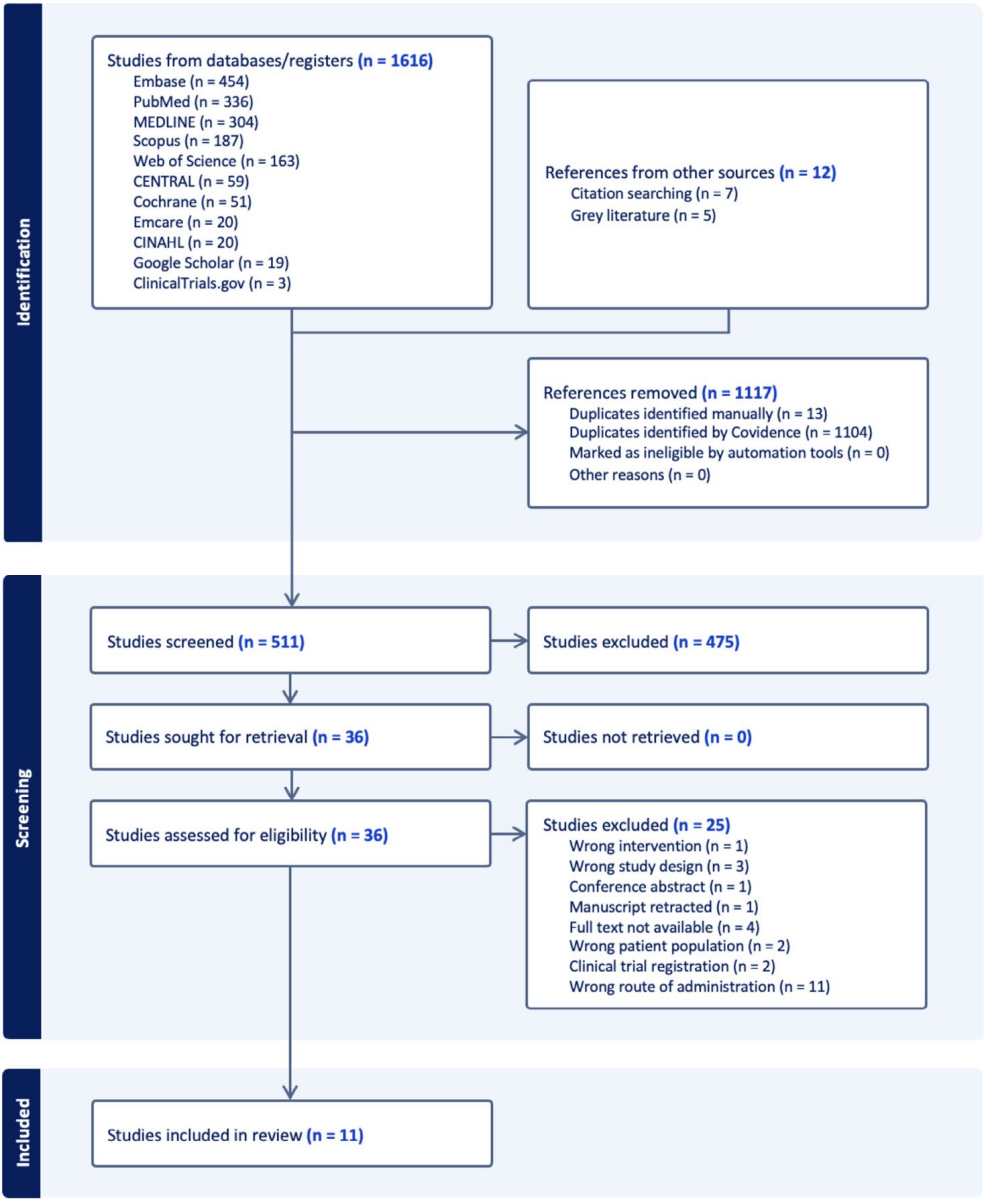
PRISMA flow diagram.

A total of 1616 unique records were identified through databases searches, with an additional 12 records retrieved from gray literature and citation tracking. After removing duplicates, 511 titles and abstracts were screened, and 36 full‐text articles were assessed for eligibility. Eleven RCTs met the inclusion criteria, encompassing 3783 participants, 1954 allocated to vaginal dinoprostone and 1829 to oral misoprostol [27, 28, 29, 30, 31, 32, 33, 34, 35, 36, 37]. The PRISMA flow diagram is presented in Figure 1. Reasons for exclusion of full‐text articles are detailed in Appendix S13 [38, 39, 40, 41, 42, 43, 44, 45, 46, 47, 48, 49, 50, 51, 52, 53, 54, 55, 56, 57, 58, 59, 60, 61].

### 3.2 Study Characteristics

A total of 11 studies involving 3783 participants were included. Study characteristics are summarized in Table 1. The included trials were conducted across diverse geographic regions, including Australia, Canada, China, Germany, Pakistan, Saudi Arabia, South Africa, Switzerland, and the United Kingdom. Study periods ranged from the late 1990s to 2020, with sample sizes ranging from 100 to 741 participants. Participant characteristics were broadly similar across studies. The mean maternal age ranged from approximately 26–30 years, and gestational age at induction was typically ≥ 38 weeks. Some studies specifically stratified dosing by parity, administering higher doses or additional applications in multiparous individuals. Nulliparous and multiparous participants were included in most studies, though the reporting of parity‐specific data varied, with some trials not clearly distinguishing parity at baseline.

**TABLE 1:** Detailed table of characteristics of included studies.

Study	Region	Study period	Sample size		Dose		Maternal age		Gestational age		Nulliparity	
			PGE1	PGE2	PGE1	PGE2	PGE1	PGE2	PGE1	PGE2	PGE1	PGE2
Hofmeyr (2001)	South Africa and United Kingdom	Not reported	346	349	200 μg dissolved in 200 mL water 20 μg 2 h for 2–3 doses then 40 μg 2 h	2 mg gel 6 h for 2 applications	27.1 ± 6.4	27.2 ± 5.9	39.7 ± 2.1	39.6 ± 2.4	NR	NR
Le Roux (2002)	South Africa	Apr 1999 to Nov 2000	120	240	50 μg for up to 4 doses	1 mg gel for up to 2 doses	28.1^c^	27.6^c^	38.3^c^	39^c^	43	101
Dällenbach (2003)	Switzerland	Sep 1999 to Apr 2001	100	100	20–40 μg dissolved in 20 mL water 2 h (2–10 doses)	2 mg gel 6 h for 2 applications	30.0 (26.0–34.0)^a^	29.5 (25.0–33.0)^a^	283 days (274–290)^a^	286 days (274–290)^a^	58	61
Matonhodze (2003)	South Africa	Not reported	174	176	200 μg dissolved in 200 mL water 20 μg 2 h for 3 doses then 40 μg 2 h	2 mg gel 6 h	27.8 ± 6.7	27.3 ± 6.2	39.9 ± 2.1	39.7 ± 2.4	65	63/174
Shetty (2004)	United Kingdom	Oct 2001 to Feb 2002	75	73	100 μg orally 4 h for up to 5 doses	3 mg tablet vaginally 6 h if needed	28.1 ± 5.9	28.6 ± 6.2	284.8 days (8.6)	284 days (9.5)	59	64
Dodd (2006)	Australia	Apr 2001 to Dec 2004	365	376	20 μg dissolved in 20 ml water 2 h for up to 6 doses	Nulliparous: 2 mg gel 6 h for 2 applications Multiparous: 1 mg gel 6 h for 2 applications	27.9 ± 5.6	28.0 ± 5.6	40.6 ± 2.0	40.4 ± 2.1	213	221
Henrich (2008)	Germany	2003–2006	112	112	25 μg, then 50 μg, then100 μg 4 h	3 mg gel 6 h	28^b^	29^b^	40.1^b^	40.6^b^	NR	NR
Rouzi (2014)	Saudi Arabia	Jan 2011 to Jul 2012	80	80	20 μg hourly for 2 doses, 30 μg hourly for 3 doses, 40 μg for 1 dose, 50 μg for 1 dose and 60 μg hourly for 4 doses	10 mg vaginal slow release insert once over 24 h	28.4 ± 5.4 (19–46)	29.1 ± 5.6 (19–49)	39.9 ± 1.8 (34–42)	39.9 ± 1.8 (35–42)	41	36
Ilyas (2016)	Pakistan	Jan 2011 to Jun 2011	50	50	50 μg 4 h for up to 3 doses	0.5 mg gel 6 h up to 2 doses	26.0 ± 3.8	25.7 ± 3.9	38.8 ± 1.1 weeks	38.9 ± 1.2 weeks	NR	NR
Wang (2016)	China	Jan to Oct 2014	212	199	20 μg hourly for 2 doses, 30 μg hourly for 3 doses, 40 μg for 1 dose (after 1.5 h), 50 μg for 1 dose (after 2 h) and 60 μg 2 h for 2 doses	Vaginal insert given “according to drug protocol”	27.8 ± 4.8 (18–39)	28.3 ± 5.6 (18–35)	39.5 ± 6.2 (36–42)	38.8 ± 7.1 (36–42)	NR	NR
Young (2020)	Canada	Apr 1999 to Dec 2000	167	172	50 μg 4 h	1–2 mg 6 h for up to 6 doses	29.1 ± 6.6	29.1 ± 5.7	40.0 ± 1.5	40.0 ± 1.5	108	107

Abbreviations: NR = nonreported; PGE1 = oral misoprostol; PGE2 = vaginal dinoprostone.

^a^ Median (IQ range: 25% percentile, 75th percentile).

^b^ Median.

^c^ Mean.

Across the 11 included RCTs, oral misoprostol (PGE1) dosing regimens varied substantially, with starting doses ranging from 20 to 100 μg and dosing intervals typically every 1–4 h. Both fixed‐dose and titrated regimens were used, with variation in maximum cumulative doses and treatment duration. Vaginal dinoprostone (PGE2) was administered in the form of 1–3 mg gels, 3 mg tablets, or 10 mg slow‐release vaginal inserts, with dosing frequencies ranging from 6‐h to single‐application protocols. The wide variation in dosing regimens may have contributed to variability in both efficacy and safety estimates. Table 1 explicitly reports, for each study, the starting dose, dosing interval, and whether a fixed or titrated dosing regimen was used.

### 3.3 Risk of Bias Assessment

Risk of bias was assessed using the Cochrane Risk of Bias 2.0 tool across five domains: randomization process, deviations from intended interventions, missing outcome data, measurement of outcomes, and selection of reported results [16]. Most studies were rated as having “some concerns,” primarily due to lack of blinding of participants, care providers, and outcome assessors. Only one study implemented double‐blinding using identical treatment packs and was rated as low risk across all domains [32].

Randomization and allocation concealment were generally well described. Ten of the 11 trials used computer‐generated randomization sequences, and most employed sealed, opaque envelopes to ensure allocation concealment. Baseline characteristics were well balanced across groups in all studies, supporting the integrity of the randomization process.

Blinding was the most common limitation. Due to the nature of the interventions, oral versus vaginal administration, blinding of participants and clinicians was not feasible in 10 studies, introducing potential performance bias, particularly, for outcomes influenced by clinical decision‐making, such as oxytocin augmentation or timing of cesarean delivery. Detection bias was also a concern for outcomes requiring subjective interpretation, such as fetal heart rate tracings and maternal side effects. Few studies reported blinding of outcome assessors, and even then, blinding was often limited to specific outcomes (e.g., cardiotocograph interpretation).

Attrition bias was low across studies, with most reporting complete or near‐complete follow‐up and consistent use of intention‐to‐treat analysis. Selective reporting was rated as “some concerns” in several trials due to the absence of pre‐registered protocols or trial registrations, limiting confirmation of outcome completeness. However, no evidence of outcome suppression or data manipulation was identified.

**FIGURE 2:**
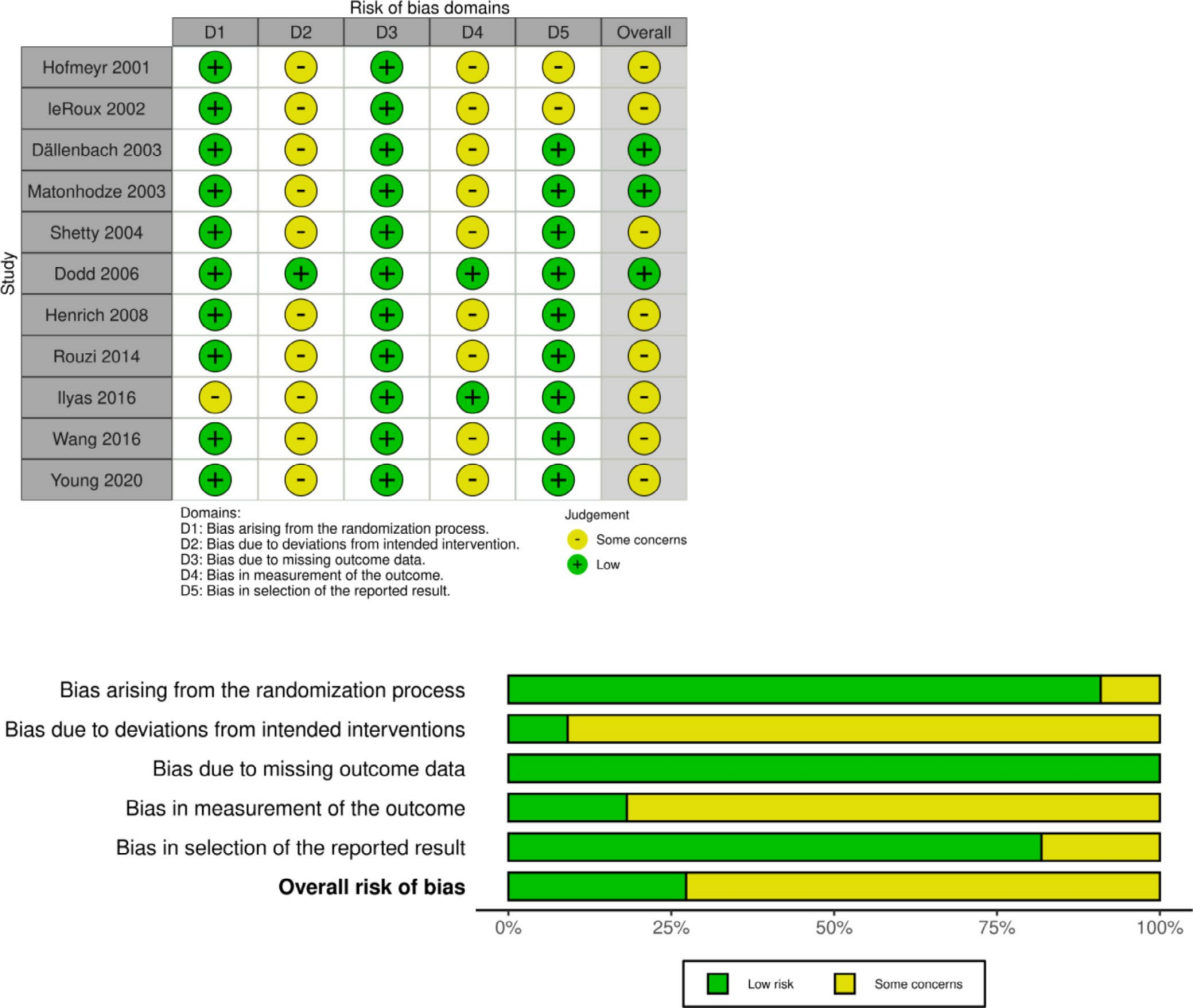
Detailed risk of bias assessment of included studies.

A visual summary is provided in Figure 2 and a detailed breakdown of risk of bias assessments for each study is presented in Appendices S14–S24.

### 3.4 Critical Outcomes

**FIGURE 3:**
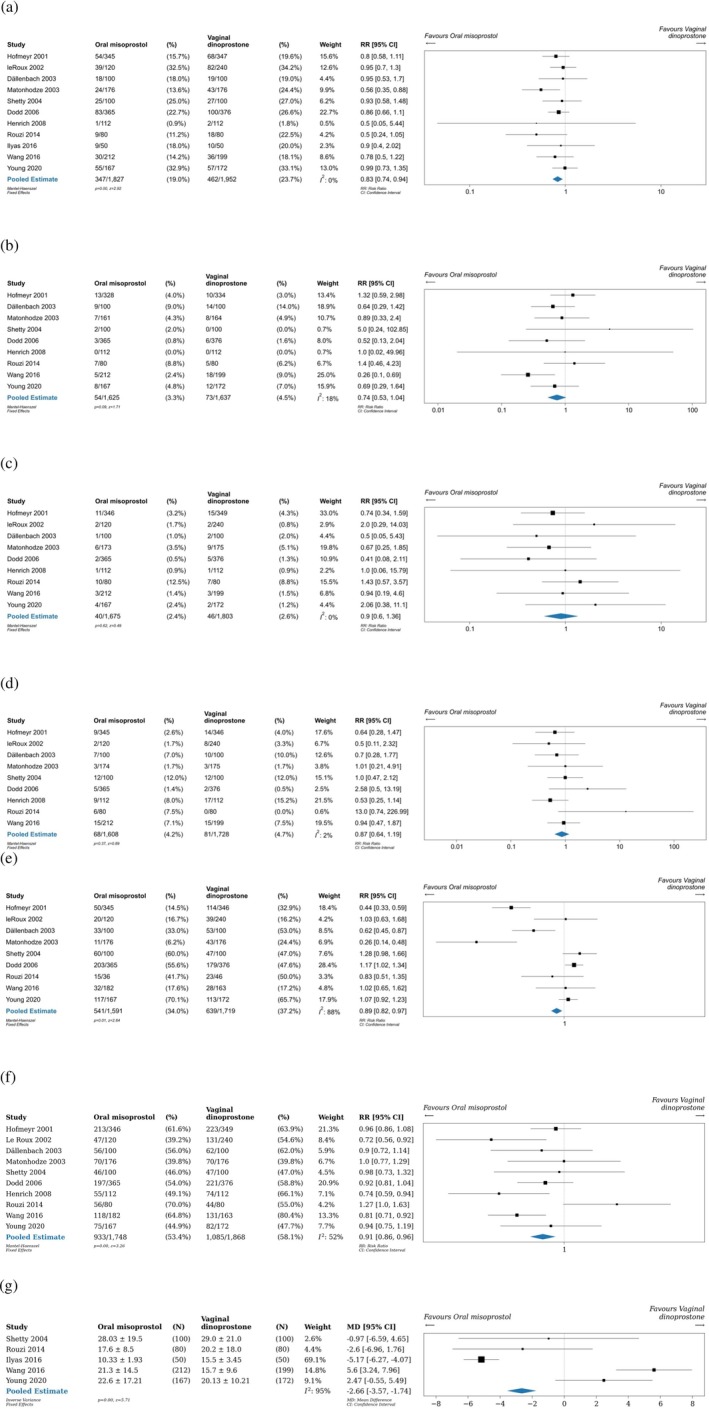
Main analysis of critical outcomes. (a) Cesarean birth, (b) uterine hyperstimulation, (c) Apgar score < 7 at 5 min, (d) admission to neonatal intensive care unit, (e) oxytocin augmentation, (f) vaginal birth within 24 h, and (g) induction to vaginal birth.

Vaginal dinoprostone was associated with an increased likelihood of cesarean birth compared with oral misoprostol, with a RR of 0.83, 95% CI 0.74–0.94, corresponding to absolute event rates of 19.0% versus 23.7% in the intervention and comparator groups, respectively. Statistical heterogeneity was low (*I* ^2^ = 23%). The certainty of evidence was high. Results are shown in Figure 3a and summarized in the GRADE Summary of Findings (Table 2).

**TABLE 2:** GRADE assessment of critical outcomes.

Certainty assessment							Summary of findings				
Participants (studies) Follow‐up	Risk of bias	Inconsistency	Indirectness	Imprecision	Publication bias	Overall certainty of evidence	Study event rates (%)			Anticipated absolute effects	
							With vaginal dinoprostone	With oral misoprostol	Relative effect (95% CI)	Risk with vaginal dinoprostone	Risk difference with oral misoprostol
1. Cesarean birth											
3779 (11 RCTs)	Not serious	Not serious	Not serious	Not serious	None	⨁⨁⨁⨁ High	462/1952 (23.7%)	347/1827 (19.0%)	RR 0.83 (0.74–0.94)	462/1952 (23.7%)	40 fewer per 1000 (from 62 fewer to 14 fewer)
2. Uterine hyperstimulation											
3262 (9 RCTs)	Not serious	Serious^a^	Not serious	Serious^b^	None	⨁⨁◯◯ Low^a^, ^b^	73/1637 (4.5%)	54/1625 (3.3%)	RR 0.74 (0.53–1.04)	73/1637 (4.5%)	12 fewer per 1000 (from 21 fewer to 2 more)
3. Apgar score < 7 at 5 min											
3478 (9 RCTs)	Not serious	Not serious	Not serious	Serious^c^	None	⨁⨁⨁◯ Moderate^c^	46/1803 (2.6%)	40/1675 (2.4%)	RR 0.90 (0.60–1.36)	46/1803 (2.6%)	3 fewer per 1000 (from 10 fewer to 9 more)
4. Admission to neonatal intensive care unit											
3336 (9 RCTs)	Not serious	Not serious	Not serious	Serious^c^	None	⨁⨁⨁◯ Moderate^c^	81/1728 (4.7%)	68/1608 (4.2%)	RR 0.87 (0.64–1.19)	81/1728 (4.7%)	6 fewer per 1000 (from 9 more to 405 more)
5. Oxytocin augmentation											
3310 (9 RCTs)	Not serious	Serious^d^	Not serious	Not serious	None	⨁⨁⨁◯ Moderate^d^	639/1719 (37.2%)	541/1591 (34.0%)	RR 0.89 (0.82–0.97)	639/1719 (37.2%)	41 fewer per 1000 (from 67 fewer to 11 fewer)
6. Vaginal birth within 24 h											
3613 (10 RCTs)	Not serious	Not serious	Not serious	Not serious	None	⨁⨁⨁⨁ High	1085/1868 (58.1%)	933/1748 (53.4%)	RR 0.91 (0.86–0.96)	581/1000 (58.1)	37 fewer per 1000 (from 21 fewer to 100 more)
7. Induction to vaginal birth interval											
1210 (5 RCTs)	Not serious	Serious^e^	Not serious	Serious^f^	None	⨁⨁◯◯ Low^e^, ^f^	601	609	—	601	MD 2.66 time (h) lower (3.57 lower to 1.74 lower)

Abbreviations: CI: confidence interval; MD: mean difference; RR: risk ratio.

^a^ Different studies reported varying rates of uterine hyperstimulation, particularly, with vaginal misoprostol, leading to inconsistent findings.

^b^ The estimates for uterine hyperstimulation were imprecise due to wide confidence intervals in some studies.

^c^ The confidence intervals for neonatal outcomes were wide in some studies, leading to imprecision.

^d^ The need for oxytocin augmentation varied significantly between studies, leading to inconsistent results.

^e^ The time to vaginal delivery varied significantly between studies, with some showing faster times for certain methods and others not.

^f^ The estimates for time to vaginal delivery were imprecise due to wide confidence intervals in some studies.

Oral misoprostol was associated with no difference in risk of uterine hyperstimulation compared with vaginal dinoprostone (RR 0.74, 95% CI 0.53–1.04), corresponding to absolute risks of 3.3% versus 4.5%, respectively. Heterogeneity was moderate (*I* ^2^ = 33%). Due to inconsistency and imprecision, the certainty of evidence was low (Figure 3b).

The 5‐min Apgar score < 7 occurred at comparable rates in both groups (RR 0.90, 95% CI 0.60–1.36) with absolute risks of 2.4% and 2.6%, respectively. With low heterogeneity (*I* ^2^ = 15%), and moderate‐certainty evidence (Figure 3c).

There was no difference in NICU admission between groups (RR 0.87, 95% CI 0.64–1.19) corresponding to absolute rates of 4.2% versus 4.7%, respectively. Heterogeneity was low (*I* ^2^ = 18%) with moderate‐certainty evidence (Figure 3d).

Oxytocin augmentation was required in 34.0% of participants receiving oral misoprostol and 37.2% receiving vaginal dinoprostone (RR 0.89, 95% CI 0.82–0.97), corresponding to 41 fewer per 1000 (from 67 fewer to 11 fewer). Heterogeneity was moderate (*I* ^2^ = 35%), with a moderate certainty of evidence (Figure 3e).

Vaginal dinoprostone was associated with a higher likelihood of vaginal birth within 24 h (RR 0.91, 95% CI 0.86–0.96), corresponding to absolute rates of 58.1% versus 53.4%. This outcome was supported by substantial heterogeneity (*I* ^2^ = 55%) and high‐certainty evidence (Figure 3f).

Vaginal dinoprostone was associated with a shorter induction‐to‐birth interval (mean difference 2.66 h, 95% CI −3.57 to −1.74). Statistical heterogeneity was moderate (*I* ^2^ = 45%). The certainty of evidence was low, downgraded for inconsistency and imprecision (Figure 3g).

### 3.5 Important Outcomes

#### 3.5.1 Effectiveness Outcomes

Vaginal birth within 48 h showed no significant difference between groups (RR 1.07, 95% CI 0.96–1.19; low certainty), corresponding to comparable absolute event rates across groups (54.8% vs. 52.6%). Instrumental birth rates were also similar (RR 1.04, 95% CI 0.88–1.24; low certainty), as were rates of postpartum hemorrhage (RR 0.91, 95% CI 0.78–1.08; low certainty). Across these outcomes, findings were consistent with no clear evidence of clinically meaningful differences between groups. Results are presented in Appendix S25 and summarized in the GRADE Summary of Findings (Appendix S28).

#### 3.5.2 Safety Outcomes

There were no significant differences between groups in non‐reassuring fetal heart tracings (RR 0.94, 95% CI 0.63–1.40; 5.7% vs. 6.0%) or 1‐min Apgar score < 7 (RR 1.05, 95% CI 0.72–1.51; 8.9% vs. 8.7%). Maternal side effects including nausea (7.8% vs. 7.8%), diarrhea (1.4% vs. 2.5%), vomiting (8.7% vs. 9.0%), shivering (18.4% vs. 17.1%), and fever (2.0% vs. 2.0%) were also similar between groups, with small and imprecise absolute differences (all low certainty).

Uterine tachysystole occurred at similar rates (8.1% in both groups; RR 0.93, 95% CI 0.73–1.20; low certainty). In contrast, uterine hypertonus was significantly less frequent with oral misoprostol (RR 0.43, 95% CI 0.25–0.75; low certainty), although absolute event rates remained low (2.5% vs. 5.8%). Meconium‐stained amniotic fluid was comparable between groups (15.3% vs. 16.0%; RR 0.95, 95% CI 0.80–1.13; low certainty).

These findings suggest similar safety profiles between groups across most outcomes, with the exception of reduced uterine hypertonus with misoprostol. Results are presented in Appendix S26 and summarized in the GRADE Summary of Findings (Appendix S28).

#### 3.5.3 Resource‐Related Outcomes

The need for tocolysis did not differ significantly between groups (RR 0.63, 95% CI 0.38–1.06; low‐certainty), with similar absolute rates observed. Analgesia use was also comparable (RR 0.97, 95% CI 0.92–1.02; low‐certainty). Overall, resource‐related outcomes were consistent between groups. Results are presented in Appendix S27 and summarized in the GRADE Summary of Findings (Appendix S28).

### 3.6 Subgroup Analyses

The exploratory subgroup analysis found that oral misoprostol may be more effective than vaginal dinoprostone in multiparous individuals (RR 1.16, 95% CI 1.01–1.34), corresponding to 108 more vaginal births per 1000. In contrast, vaginal dinoprostone appeared more effective in nulliparous individuals (RR 0.86, 95% CI 0.68–1.09), although this difference was not statistically significant. The test for interaction yielded a *p*‐value of 0.78, suggesting that chance is a very likely explanation for the apparent effect modification. These findings should be interpreted cautiously, as the analyses were exploratory, underpowered for interaction testing, and not intended to establish definitive subgroup effects. Detailed results are presented in Appendices S29–S31, with GRADE assessments in Appendix S32.

The ICEMAN tool was applied only to assess the credibility of parity as an effect modifier for the outcome of vaginal birth within 24 h, as outlined in Appendix S33. According to the ICEMAN assessment, the credibility of parity as an effect modifier for vaginal birth within 24 h was judged to be low. The analysis was primarily based on within‐trial comparisons, and the effect modification was mostly similar across studies. However, several limitations reduced credibility. The interaction was not statistically significant, the number of trials was small, the direction of effect modification was not hypothesized a priori, and a fixed‐effect model was used rather than a random‐effects model. Furthermore, while the analysis included data from multiple RCTs, there was some inconsistency, with at least one trial in the nulliparous subgroup showing an effect in the opposite direction.

When stratified by country income level, vaginal dinoprostone was more effective in both high‐income (RR 0.93, 95% CI 0.86–1.00) and low‐/middle‐income countries (RR 0.87, 95% CI 0.80–0.96). As no effect modification was observed between these subgroups, the ICEMAN tool was not applied.

### 3.7 Sensitivity Analysis

Sensitivity analyses were performed by excluding studies at high risk of bias to assess the robustness of the findings. These analyses are summarized in Appendix S34.

For cesarean birth, the pooled effect estimate remained stable when restricted to low‐risk‐of‐bias studies (RR 0.84, 95% CI 0.76–0.93), closely matching the overall estimate (RR 0.83, 95% CI 0.74–0.94), with low heterogeneity (*I* ^2^ = 19%).

For uterine hyperstimulation, the estimate remained consistent (RR 0.72, 95% CI 0.50–1.02) compared to the full analysis (RR 0.74, 95% CI 0.53–1.04), although the result remained statistically nonsignificant.

For vaginal birth within 24 h, the effect favoring vaginal dinoprostone persisted in the sensitivity analysis (RR 0.91, 95% CI 0.85–0.97), consistent with the main analysis (RR 0.92, 95% CI 0.86–0.98), with moderate heterogeneity (*I* ^2^ = 50%). The induction‐to‐birth interval also remained significantly shorter with vaginal dinoprostone (mean difference −2.66 h, 95% CI −3.57 to −1.74), with moderate heterogeneity (*I* ^2^ = 45%).

Other outcomes, including Apgar score < 7 at 5 min, NICU admissions, oxytocin augmentation, and maternal side effects, showed no meaningful changes in direction or magnitude of effect. Across all outcomes, no reversal in effect direction was observed, and heterogeneity remained within acceptable limits. These findings confirm that the primary conclusions are robust and not driven by lower‐quality studies or analytic assumptions.

## 4 Discussion

### 4.1 Principal Findings

This systematic review and meta‐analysis of 11 RCTs comparing oral misoprostol with vaginal dinoprostone for labor induction found that oral misoprostol is associated with a reduced risk of cesarean birth and is probably associated with a reduced need for oxytocin augmentation. Vaginal dinoprostone was associated with a higher likelihood of vaginal birth within 24 h and is probably associated with a shorter induction‐to‐birth interval. Maternal and neonatal safety outcomes, including uterine hyperstimulation, low Apgar scores, and NICU admissions, were comparable between groups. Although vaginal dinoprostone may expedite delivery, this benefit may be accompanied by a higher likelihood of cesarean birth. Overall heterogeneity was low to moderate, suggesting reasonable consistency across studies despite clinical variability.

Subgroup analyses suggested potential differences by parity, with oral misoprostol showing a trend toward greater effectiveness among multiparous individuals and vaginal dinoprostone among nulliparous individuals. However, the test for interaction was not statistically significant, and the credibility of parity as an effect modifier for vaginal birth within 24 h was rated low using the ICEMAN tool. Differences by country income level were inconsistent and showed no evidence of statistical interaction; therefore, the ICEMAN tool was not applied to this subgroup. These findings lend support to considering oral misoprostol in carefully selected populations and settings.

### 4.2 Comparison With Previous Studies

The findings of this review are broadly consistent with prior systematic reviews and meta‐analyses, which have demonstrated that oral misoprostol is associated with a reduced risk of cesarean delivery, whereas vaginal dinoprostone may be more effective in expediting vaginal birth within 24 h [9, 10, 11, 12, 13, 14, 15]. However, earlier reviews frequently pooled across heterogeneous prostaglandin formulations and routes of administration, thereby limiting the interpretability of route‐specific effects. Moreover, many lacked formal assessments of risk of bias, certainty of evidence, or effect modification.

In contrast, the present review offers a focused comparison between oral misoprostol and vaginal dinoprostone, thereby eliminating confounding introduced by mixed‐route analyses. It prioritizes outcomes of direct clinical relevance, such as mode of delivery, neonatal morbidity, and resource utilization, rather than surrogate endpoints such as time to delivery alone. Furthermore, this review incorporated rigorous methodological approaches, including duplicate independent review, risk of bias assessment using the Cochrane RoB 2.0 tool [16], certainty of evidence grading via the GRADE framework [17], and evaluation of subgroup effect credibility using the ICEMAN tool [18].

Notably, while previous reviews have emphasized the advantage of vaginal dinoprostone in achieving vaginal birth within 24 h, they have often failed to contextualize this benefit alongside its association with higher cesarean rates [7, 9, 10]. The present findings clarify this trade‐off and underscore the importance of considering both efficacy and safety when selecting an induction agent. By integrating subgroup and sensitivity analyses, this review also provides insight into potential effect modifiers, such as parity and healthcare setting, that were either absent or insufficiently explored in earlier syntheses. These contributions enhance the clinical applicability and policy relevance of the findings.

### 4.3 Strengths and Limitations

This systematic review and meta‐analysis has several methodological strengths. It is the first systematic review to focus exclusively on the comparison between oral misoprostol and vaginal dinoprostone, thereby eliminating heterogeneity introduced by pooling across different prostaglandin formulations or routes of administration. The review was conducted in accordance with a prospectively registered protocol and adhered to rigorous methodological standards, including comprehensive database searches, duplicate independent screening and data extraction, and formal risk of bias assessment [16].

The analysis prioritized patient‐important outcomes, such as mode of delivery, neonatal morbidity, and resource utilization, over surrogate endpoints like time to delivery. Certainty of evidence was evaluated using the GRADE framework [17], and the credibility of subgroup effects was assessed using the ICEMAN tool [18]. Sensitivity analyses were conducted to evaluate the robustness of findings, and subgroup analyses were used to explore potential effect modifiers, including parity and healthcare setting, enhancing the clinical applicability of the results.

Nonetheless, several limitations should be acknowledged. Clinical heterogeneity was present across included trials, particularly, in dosing regimens and formulations of both oral misoprostol and vaginal dinoprostone. Most studies were unblinded due to the nature of the interventions, introducing potential performance and detection bias, especially for outcomes requiring clinical judgment. Definitions of key outcomes, such as uterine hyperstimulation, varied across studies, contributing to inconsistency and imprecision in pooled estimates. Additionally, long‐term maternal and neonatal outcomes were not reported, limiting the ability to assess the broader implications of induction method selection.

### 4.4 Clinical Implications

The findings of this review suggest that oral misoprostol may be considered as an option for labor induction, particularly, when balancing potential reductions in cesarean birth and need for oxytocin augmentation against a lower likelihood of achieving vaginal birth within 24 h and a longer induction‐to‐birth interval compared with vaginal dinoprostone.

Given the comparable maternal and neonatal safety profiles observed between groups, the choice of induction method may reasonably be guided by clinical priorities, including urgency of delivery, resource availability, and patient preferences.

Importantly, the substantial variability in oral misoprostol dosing regimens across studies, including differences in starting dose, administration interval, and use of fixed versus titrated protocols, limits the ability to define an optimal regimen and should be carefully considered when applying these findings to clinical practice. Consistent with this, statistical heterogeneity ranged from low to moderate across outcomes, with higher heterogeneity observed for certain time‐dependent outcomes, suggesting variability in treatment effects across study settings and protocols, which may limit the generalizability of pooled estimates.

In the absence of clear evidence supporting effect modification by parity or setting, these findings do not support tailoring induction method based solely on subgroup characteristics; however, individual patient factors and clinical context remain important considerations. Shared decision‐making should incorporate individual risk profiles, anticipated delivery timelines, and values related to mode of birth and intervention intensity.

### 4.5 Future Research

Further research is needed to address limitations identified in the current evidence base. RCTs directly comparing oral misoprostol and vaginal dinoprostone using standardized dosing regimens are needed to establish optimal protocols. Harmonization of outcome definitions, particularly for uterine hyperstimulation, and consistent reporting practices are essential to improve comparability across studies.

Longitudinal studies evaluating maternal and neonatal outcomes beyond the immediate peripartum period, including postpartum recovery, breastfeeding success, and early childhood development, are warranted to better understand the long‐term implications of induction strategies. Additionally, greater emphasis on patient‐centered outcomes, such as maternal satisfaction, perceived control, and pain experience, would enhance the alignment of clinical care with patient values.

Future systematic reviews should consider individual participant data meta‐analyses to enable exploration of effect modifiers such as parity, body mass index, and cervical status. This approach would facilitate more precise estimates of treatment effects across clinically relevant subgroups and support the development of personalized induction strategies.

In conclusion, oral misoprostol is associated with a reduced risk of cesarean birth and is probably associated with a reduced need for oxytocin augmentation. Vaginal dinoprostone is associated with a higher likelihood of vaginal birth within 24 h and is probably associated with a shorter induction‐to‐birth interval. Maternal and neonatal safety outcomes appear comparable between groups; however, these findings should be interpreted in the context of variability in dosing regimens and the presence of some low‐certainty evidence. Oral misoprostol may be considered in carefully selected populations and settings.

## Author Contributions

**Tiffany Yeretsian:** conceptualization, methodology, validation, visualization, writing – review and editing, writing – original draft, project administration, software, formal analysis, investigation, data curation, resources. **Hussain Mogharbel:** conceptualization, methodology, writing – review and editing. **Rohan D'Souza:** writing – review and editing, supervision, conceptualization, methodology. **Kristian Thorlund:** writing – review and editing, supervision. **Abirami Kirubarajan:** writing – review and editing. **Rizwana Ashraf:** formal analysis, data curation, writing – review and editing. **Rayyan Rozzah:** conceptualization, methodology, writing – review and editing.

## Funding

The authors have nothing to report.

## Ethics Statement

The authors have nothing to report.

## Consent

The authors have nothing to report.

## Conflicts of Interest

Rohan D'Souza reports grants from the International Vasa Previa Foundation, the Perinatal Society of Australia and New Zealand, the Canadian Institutes for Health Research, Heart and Stroke Foundation, Brain Canada, Public Health Agency of Canada, Juravinski Research Institute, and Hamilton Academic Health Sciences Organization during the conduct of the study, and personal fees from Ferring, Sanofi, and Pfizer outside the submitted work. He is lead investigator on the Outcome Reporting in Obstetric Studies project, funded through a Tier‐2 Canada Research Chair (#CRC‐2021‐00337). The other authors declare no conflicts of interest.

## Supporting information


**Appendix S1:** Ovid MEDLINE.
**Appendix S2:** EMBASE.
**Appendix S3:** PUBMED.
**Appendix S4:** CENTRAL 19.
**Appendix S5:** SCOPUS.
**Appendix S6:** ClinicalTrials.Gov
**Appendix S7:** Web of Science.
**Appendix S8:** CINHAL EBSCOHost.
**Appendix S9:** Google scholar.
**Appendix S10:** Emcare.
**Appendix S11:** Cochrane.
**Appendix S12:** Definitions of outcomes.
**Appendix S13:** Table of excluded full‐text articles.
**Appendix S14:** Hofmeyr 2001.
**Appendix S15:** le Roux 2002.
**Appendix S16:** Dällenbach 2003.
**Appendix S17:** Matonhodze 2003.
**Appendix S18:** Shetty 2004.
**Appendix S19:** Dodd 2006.
**Appendix S20:** Henrich 2008.
**Appendix S21:** Rouzi 2014.
**Appendix S22:** Ilyas 2016.
**Appendix S23:** Wang 2016.
**Appendix S24:** Young 2020.
**Appendix S25:** Important outcomes related to effectiveness.
**Appendix S26:** Important outcomes related to safety.
**Appendix S27:** Important outcome related to resource use.
**Appendix S28:** Core GRADE assessment for important outcomes.
**Appendix S29:** Vaginal birth within 24 h‐stratified by parity.
**Appendix S30:** Vaginal birth within 24 h stratified by country income level.
**Appendix S31:** Cesarean birth stratified by country income level.
**Appendix S32:** Core GRADE assessment for subgroup analyses.
**Appendix S33:** ICEMAN judgments.
**Appendix S34:** Sensitivity analysis of outcomes.
**Appendix S35:** PRISMA 2020 checklist.

## Data Availability

The data that supports the findings of this study are available in the Supporting Information of this article.
