# Supplementary material for: Oral Misoprostol Versus Vaginal Dinoprostone for Labor Induction: A Systematic Review and Meta‐Analysis
Source: J Obstet Gynaecol Res. 2026 Jul 24;52(8):e70369. doi: 10.1111/jog.70369 (PMC13400899; doi:10.1111/jog.70369)
Supplement: Supplementary file 1 — Appendix S1: Ovid MEDLINE. Appendix S2: EMBASE. Appendix S3: PUBMED. Appendix S4: CENTRAL 19. Appendix S5: SCOPUS. Appendix S6: ClinicalTrials.Gov Appendix S7: Web of Science. Appendix S8: CINHAL EBSCOHost. Appendix S9: Google scholar. Appendix S10: Emcare. Appendix S11: Cochrane. Appendix S12: Definitions of outcomes. Appendix S13: Table of excluded full‐text articles. Appendix S14: Hofmeyr 2001. Appendix S15: le Roux 2002. Appendix S16: Dällenbach 2003. Appendix S17: Matonhodze 2003. Appendix S18: Shetty 2004. Appendix S19: Dodd 2006. Appendix S20: Henrich 2008. Appendix S21: Rouzi 2014. Appendix S22: Ilyas 2016. Appendix S23: Wang 2016. Appendix S24: Young 2020. Appendix S25: Important outcomes related to effectiveness. Appendix S26: Important outcomes related to safety. Appendix S27: Important outcome related to resource use. Appendix S28: Core GRADE assessment for important outcomes. Appendix S29: Vaginal birth within 24 h‐stratified by parity. Appendix S30: Vaginal birth within 24 h stratified by country income level. Appendix S31: Cesarean birth stratified by country income level. Appendix S32: Core GRADE assessment for subgroup analyses. Appendix S33: ICEMAN judgments. Appendix S34: Sensitivity analysis of outcomes. Appendix S35: PRISMA 2020 checklist. [file JOG-52-0-s001.docx]

Supporting Information

Oral Misoprostol Versus Vaginal Dinoprostone for Labour Induction: A Systematic Review and Meta-Analysis. Yeretsian T, Mogharbel H, Rozzah R, Ashraf R, Kirubarajan A, Thorlund K, D’Souza R.

Table of Contents

[Search Strategy 4](#_Toc235544006)

[Appendix S1. Ovid MEDLINE® 4](#_Toc235544007)

[Appendix S2. EMBASE 5](#_Toc235544008)

[Appendix S3. PUBMED 6](#_Toc235544009)

[Appendix S4. CENTRAL 19 7](#_Toc235544010)

[Appendix S5. SCOPUS 7](#_Toc235544011)

[Appendix S6. ClinicalTrials.Gov 7](#_Toc235544012)

[Appendix S7. Web of Science 8](#_Toc235544013)

[Appendix S8. CINHAL EBSCOHost 8](#_Toc235544014)

[Appendix S9. Google scholar 8](#_Toc235544015)

[Appendix S10. Emcare 8](#_Toc235544016)

[Appendix S11. Cochrane 9](#_Toc235544017)

[Definition of Outcomes 10](#_Toc235544018)

[Appendix S12. Definitions of outcomes 10](#_Toc235544019)

[Excluded studies 11](#_Toc235544020)

[Appendix S13. Table of excluded full-text articles 11](#_Toc235544021)

[Risk of Bias Assessments 12](#_Toc235544022)

[Appendix S14. Hofmeyr 2001 12](#_Toc235544023)

[Appendix S15. le Roux 2002 14](#_Toc235544024)

[Appendix S16. Dällenbach 2003 16](#_Toc235544025)

[Appendix S17. Matonhodze 2003 19](#_Toc235544026)

[Appendix S18. Shetty 2004 22](#_Toc235544027)

[Appendix S19. Dodd 2006 24](#_Toc235544028)

[Appendix S20. Henrich 2008 26](#_Toc235544029)

[Appendix S21. Rouzi 2014 29](#_Toc235544030)

[Appendix S22. Ilyas 2016 32](#_Toc235544031)

[Appendix S23. Wang 2016 35](#_Toc235544032)

[Appendix S24. Young 2020 37](#_Toc235544033)

[Pairwise Meta-Analysis of Important Outcomes 41](#_Toc235544034)

[Appendix S25. Important Outcomes Related to Effectiveness 41](#_Toc235544035)

[Appendix S26. Important Outcomes Related to Safety 41](#_Toc235544036)

[Appendix S27. Important Outcome Related to Resource Use 43](#_Toc235544037)

[Core GRADE Assessment for Important Outcomes 44](#_Toc235544038)

[Appendix S28. Core GRADE assessment for important outcomes 44](#_Toc235544039)

[Subgroup pairwise analysis (Outcome/Subgroup) 48](#_Toc235544040)

[Appendix S29. Vaginal Birth Within 24 Hours-Stratified by Parity 48](#_Toc235544041)

[Appendix S30. Vaginal Birth Within 24 Hours Stratified by Country Income Level 48](#_Toc235544042)

[Appendix S31. Cesarean Birth Stratified by Country Income Level 48](#_Toc235544043)

[Core GRADE Assessment for Subgroup Analyses 49](#_Toc235544044)

[Appendix S32. Core GRADE Assessment for Subgroup Analyses 49](#_Toc235544045)

[ICEMAN Tool Judgements 50](#_Toc235544046)

[Appendix S33. ICEMAN judgements 50](#_Toc235544047)

[Sensitivity Analysis 51](#_Toc235544048)

[Appendix S34. Sensitivity Analysis of Outcomes 51](#_Toc235544049)

**APPENDIX S35:** PRISMA 2020 Checklist 52

# **Search Strategy**

## Appendix S1. Ovid MEDLINE®

<https://libaccess.mcmaster.ca/login?url=http://ovidsp.ovid.com/ovidweb.cgi?T=JS&NEWS=N&PAGE=main&SHAREDSEARCHID=32wNxUzMoK6gKeUjlR5rl9ftt81mqEl7IURQTEpzGKGNIe2xalCxibpnfg4AAFkzZ>

| **#** | **Query** | **Results from 27 May 2025** |
| --- | --- | --- |
| 1 | labor.mp. | 151,366 |
| 2 | labour.mp. | 40,457 |
| 3 | 1 or 2 | 179,401 |
| 4 | induction.mp. | 657,913 |
| 5 | induce.mp. | 555,917 |
| 6 | 4 or 5 | 1,135,792 |
| 7 | 3 and 6 | 12,506 |
| 8 | uterine contraction.mp. | 8,819 |
| 9 | cervical.mp. | 324,103 |
| 10 | ripening.mp. | 16,520 |
| 11 | cervical ripening.mp. | 2,657 |
| 12 | cervical ripening agent.mp. | 63 |
| 13 | 8 or 9 or 10 or 11 or 12 | 345,905 |
| 14 | misoprostol.mp. | 6,453 |
| 15 | prostaglandin E1.mp. | 6,985 |
| 16 | PGE1.mp. | 5,683 |
| 17 | misodel.mp. | 14 |
| 18 | cytotec.mp. | 148 |
| 19 | 14 or 15 or 16 or 17 or 18 | 15,888 |
| 20 | 13 and 19 | 1,303 |
| 21 | dinoprostone.mp. | 30,150 |
| 22 | dinoprost.mp. | 11,847 |
| 23 | prostaglandin E2.mp. | 26,997 |
| 24 | PGE2.mp. | 29,445 |
| 25 | propess.mp. | 74 |
| 26 | cervidil.mp. | 25 |
| 27 | prepidil.mp. | 61 |
| 28 | prostin.mp. | 137 |
| 29 | 21 or 22 or 23 or 24 or 25 or 26 or 27 or 28 | 58,682 |
| 30 | randomized controlled trial.pt. | 639,115 |
| 31 | controlled clinical trial.pt. | 95,683 |
| 32 | randomized.ab. | 693,583 |
| 33 | placebo.ab. | 258,916 |
| 34 | drug therapy.fs. | 2,816,205 |
| 35 | randomly.ab. | 460,836 |
| 36 | trial.ab. | 755,742 |
| 37 | groups.ab. | 2,857,011 |
| 38 | 30 or 31 or 32 or 33 or 34 or 35 or 36 or 37 | 6,299,304 |
| 39 | exp animals/ not humans.sh. | 5,340,475 |
| 40 | 38 not 39 | 5,522,033 |
| 41 | 7 and 20 and 29 and 40 | 153 |

## Appendix S2. EMBASE

<https://libaccess.mcmaster.ca/login?url=http://ovidsp.ovid.com/ovidweb.cgi?T=JS&NEWS=N&PAGE=main&SHAREDSEARCHID=7REOLXUPA4vlBd5xkBWw7PAMs356ZbTg7yBUVuBhw1i9Be9IqnJmxJxu4kfEQnCDC>

| **#** | **Query** | **Results from 27 May 2025** |
| --- | --- | --- |
| 1 | labor.mp. | 200,514 |
| 2 | labour.mp. | 56,751 |
| 3 | 1 or 2 | 236,293 |
| 4 | induction.mp. | 878,961 |
| 5 | induce.mp. | 719,756 |
| 6 | 4 or 5 | 1,492,943 |
| 7 | 3 and 6 | 26,889 |
| 8 | uterine contraction.mp. | 2,361 |
| 9 | cervical.mp. | 421,681 |
| 10 | ripening.mp. | 17,484 |
| 11 | cervical ripening.mp. | 3,405 |
| 12 | cervical ripening agent.mp. | 113 |
| 13 | 8 or 9 or 10 or 11 or 12 | 437,333 |
| 14 | misoprostol.mp. | 15,752 |
| 15 | prostaglandin E1.mp. | 23,732 |
| 16 | PGE1.mp. | 7,590 |
| 17 | misodel.mp. | 26 |
| 18 | cytotec.mp. | 1,403 |
| 19 | 14 or 15 or 16 or 17 or 18 | 39,717 |
| 20 | 13 and 19 | 2,368 |
| 21 | dinoprostone.mp. | 1,324 |
| 22 | dinoprost.mp. | 477 |
| 23 | prostaglandin E2.mp. | 73,093 |
| 24 | PGE2.mp. | 41,781 |
| 25 | propess.mp. | 300 |
| 26 | cervidil.mp. | 152 |
| 27 | prepidil.mp. | 258 |
| 28 | prostin.mp. | 893 |
| 29 | 21 or 22 or 23 or 24 or 25 or 26 or 27 or 28 | 79,114 |
| 30 | (Randomized controlled trial/ or controlled clinical study/ or random$.ti,ab. or randomization/ or intermethod comparison/ or placebo.ti,ab. or (compare or compared or comparison).ti. or ((evaluated or evaluate or evaluating or assessed or assess) and (compare or compared or comparing or comparison)).ab. or (open adj label).ti,ab. or ((double or single or doubly or singly) adj (blind or blinded or blindly)).ti,ab. or double blind procedure/ or parallel group$1.ti,ab. or (crossover or cross over).ti,ab. or ((assign$ or match or matched or allocation) adj5 (alternate or group$1 or intervention$1 or patient$1 or subject$1 or participant$1)).ti,ab. or (assigned or allocated).ti,ab. or (controlled adj7 (study or design or trial)).ti,ab. or (volunteer or volunteers).ti,ab. or human experiment/ or trial.ti.) not (((random$ adj sampl$ adj7 ("cross section$" or questionnaire$1 or survey$ or database$1)).ti,ab. not (comparative study/ or controlled study/ or randomi?ed controlled.ti,ab. or randomly assigned.ti,ab.)) or (Cross-sectional study/ not (randomized controlled trial/ or controlled clinical study/ or controlled study/ or randomi?ed controlled.ti,ab. or control group$1.ti,ab.)) or (((case adj control$) and random$) not randomi?ed controlled).ti,ab. or (Systematic review not (trial or study)).ti. or (nonrandom$ not random$).ti,ab. or "Random field$".ti,ab. or (random cluster adj3 sampl$).ti,ab. or ((review.ab. and review.pt.) not trial.ti.) or ("we searched".ab. and (review.ti. or review.pt.)) or "update review".ab. or (databases adj4 searched).ab. or ((rat or rats or mouse or mice or swine or porcine or murine or sheep or lambs or pigs or piglets or rabbit or rabbits or cat or cats or dog or dogs or cattle or bovine or monkey or monkeys or trout or marmoset$1).ti. and animal experiment/) or (Animal experiment/ not (human experiment/ or human/))) | 6,319,010 |
| 31 | 7 and 20 and 29 and 30 | 243 |

## Appendix S3. PUBMED

<https://pubmed.ncbi.nlm.nih.gov/?term=%28%28labor%29+OR+%28labour%29%29+AND+%28%28induction%29+OR+%28induce%29%29+AND+%28%28uterine+contraction%29+OR+%28cervical%29+OR+%28ripening%29+OR+%28cervical+ripening%29+OR+%28cervical+ripening+agent%29%29+AND+%28%28misoprostol%29+OR+%28prostaglandin+E1%29+OR+%28PGE1%29+OROR+%28misodel%29+OR+%28Cytotec%29%29+AND+%28%28dinoprostone%29+OR+%28dinoprost%29+OR+%28prostaglandin+E2%29+OR+%28PGE2%29+OR+%28propess%29+OR+%28cervidil%29+OR+%28prepidil%29+OR+%28prostin%29%29+AND+%28%28randomized+controlled+trial%5Bpt%5D%29+OR+%28controlled+clinical+trial%5Bpt%5D%29+OR+%28randomized%5Btiab%5D+OR+randomised%5Btiab%5D%29+OR+%28placebo%5Btiab%5D%29+OR+%28drug+therapy%5Bsh%5D%29+OR+%28randomly%5Btiab%5D%29+OR+%28trial%5Btiab%5D%29+OR+%28groups%5Btiab%5D%29%29+NOT+%28animals%5Bmh%5D+NOT+humans%5Bmh%5D%29&sort=date&size=200>

((labor) OR (labour)) AND ((induction) OR (induce)) AND ((uterine contraction) OR (cervical) OR (ripening) OR (cervical ripening) OR (cervical ripening agent)) AND ((misoprostol) OR (prostaglandin E1) OR (PGE1) OROR (misodel) OR (Cytotec)) AND ((dinoprostone) OR (dinoprost) OR (prostaglandin E2) OR (PGE2) OR (propess) OR (cervidil) OR (prepidil) OR (prostin)) AND ((randomized controlled trial[pt]) OR (controlled clinical trial[pt]) OR (randomized[tiab] OR randomised[tiab]) OR (placebo[tiab]) OR (drug therapy[sh]) OR (randomly[tiab]) OR (trial[tiab]) OR (groups[tiab])) NOT (animals[mh] NOT humans[mh])

## Appendix S4. CENTRAL 19

<https://www-cochranelibrary-com.libaccess.lib.mcmaster.ca/advanced-search/search-manager?p_p_id=58_INSTANCE_MODAL&p_p_lifecycle=0&p_p_state=normal&saveLastPath=false&_58_INSTANCE_MODAL_redirect=%2Fadvanced-search%2Fsearch-manager>

(((labor) OR (labour)) AND ((induction) OR (induce)) AND ((uterine contraction) OR (cervical) OR (ripening) OR (cervical ripening) OR (cervical ripening agent)) AND ((misoprostol) OR (prostaglandin E1) OR (PGE1) OR (misodel) OR (Cytotec) OR (oral misoprostol)) AND ((dinoprostone) OR (dinoprost) OR (prostaglandin E2) OR (PGE2) OR (propess) OR (cervidil) OR (prepidil) OR (prostin)):ti,ab,kw) AND (RCT OR "randomized controlled trial"):ti,ab,kw

## Appendix S5. SCOPUS

<https://www.scopus.com/results/results.uri?sort=plf-f&src=s&sid=0aff6b3cf35a02987e28a29dc1b9f2e9&sot=b&sdt=b&sl=441&s=TITLE-ABS-KEY%28%28%22labor%22+OR+%22labour%22%29+AND+%28%22induction%22+OR+%22induce%22%29+AND+%28%22uterine+contraction%22+OR+%22cervical%22+OR+%22ripening%22+OR+%22cervical+ripening%22+OR+%22cervical+ripening+agent%22%29+AND+%28%22misoprostol%22+OR+%22prostaglandin+E1%22+OR+%22PGE1%22+OR+%22misodel%22+OR+%22Cytotec%22%29+AND+%28%22dinoprostone%22+OR+%22dinoprost%22+OR+%22prostaglandin+E2%22+OR+%22PGE2%22+OR+%22propess%22+OR+%22cervidil%22+OR+%22prepidil%22+OR+%22prostin%22%29+AND+%28%22randomized+controlled+trial%22+OR+%22RCT%22+OR+%22randomized+trial%22%29%29&origin=savedSearchNewOnly&txGid=40bacb49c1d8c556d201ccaa5497182a&sessionSearchId=0aff6b3cf35a02987e28a29dc1b9f2e9&limit=10>

("labor" OR "labour") AND ("induction" OR "induce") AND ("uterine contraction" OR "cervical" OR "ripening" OR "cervical ripening" OR "cervical ripening agent") AND ("misoprostol" OR "prostaglandin E1" OR "PGE1" OR "misodel" OR "Cytotec") AND ("dinoprostone" OR "dinoprost" OR "prostaglandin E2" OR "PGE2" OR "propess" OR "cervidil" OR "prepidil" OR "prostin") AND ("randomized controlled trial" OR "RCT" OR "randomized trial")

## Appendix S6. ClinicalTrials.Gov

<https://clinicaltrials.gov/expert-search?term=(%22labor%22%20OR%20%22labour%22)%20AND%0A(%22induction%22%20OR%20%22induce%22)%20AND%0A(%22uterine%20contraction%22%20OR%20%22cervical%22%20OR%20%22ripening%22%20OR%20%22cervical%20ripening%22%20OR%20%22cervical%20ripening%20agent%22)%20AND%0A(%22misoprostol%22%20OR%20%22prostaglandin%20E1%22%20OR%20%22PGE1%22%20OR%20%22misodel%22%20OR%20%22Cytotec%22)%20AND%0A(%22dinoprostone%22%20OR%20%22dinoprost%22%20OR%20%22prostaglandin%20E2%22%20OR%20%22PGE2%22%20OR%20%22propess%22%20OR%20%22cervidil%22%20OR%20%22prepidil%22%20OR%20%22prostin%22)%20AND%0A(%22randomized%20controlled%20trial%22%20OR%20%22RCT%22%20OR%20%22randomized%20trial%22)>

("labor" OR "labour") AND ("induction" OR "induce") AND ("uterine contraction" OR "cervical" OR "ripening" OR "cervical ripening" OR "cervical ripening agent") AND ("misoprostol" OR "prostaglandin E1" OR "PGE1" OR "misodel" OR "Cytotec") AND ("dinoprostone" OR "dinoprost" OR "prostaglandin E2" OR "PGE2" OR "propess" OR "cervidil" OR "prepidil" OR "prostin") AND ("randomized controlled trial" OR "RCT" OR "randomized trial")

## Appendix S7. Web of Science

<https://www.webofscience.com/wos/alldb/summary/bff8d145-c807-4fc5-b09f-e8707b2a9f33-011bfee1f7/relevance/1>

TS=(labor OR labour) AND TS=(induction OR induce) AND TS=(uterine contraction OR cervical OR ripening OR "cervical ripening" OR "cervical ripening agent") AND TS=(misoprostol OR "prostaglandin E1" OR PGE1 OR misodel OR Cytotec) AND TS=(dinoprostone OR dinoprost OR "prostaglandin E2" OR PGE2 OR propess OR cervidil OR prepidil OR prostin) AND TS=("randomized controlled trial" OR RCT OR "randomized trial")

## Appendix S8. CINHAL EBSCOHost

<http://libaccess.mcmaster.ca/login?url=https://search.ebscohost.com/login.aspx?direct=true&db=cin20&bquery=(labor+OR+labour)+AND+(induction+OR+induce+OR+induced+OR+iol)+AND+(uterine+contraction+OR+cervical+OR+ripening+OR+cervical+ripening+OR+cervical+ripening+agent)+AND+(misoprostol+OR+prostaglandin+E1+OR+PGE1+OR+misodel+OR+cytotec)+AND+(dinoprostone+OR+prostaglandin+E2+OR+PGE2+OR+process+OR+cervidil+OR+prepidil+OR+pristine)+AND+(randomized+controlled+trials+OR+rtc+OR+randomised+control+trials)&type=1&searchMode=Standard&site=ehost-live>

("labor" OR "labour") AND ("induction" OR "induce") AND

("uterine contraction" OR "cervical" OR "ripening" OR "cervical ripening" OR "cervical ripening agent") AND

("misoprostol" OR "prostaglandin E1" OR "PGE1" OR "misodel" OR "Cytotec") AND

("dinoprostone" OR "dinoprost" OR "prostaglandin E2" OR "PGE2" OR "propess" OR "cervidil" OR "prepidil" OR "prostin") AND

("randomized controlled trial" OR "RCT" OR "randomized trial")

## Appendix S9. Google scholar

<https://scholar.google.ca/scholar?start=0&q=allintitle:+(%22labor%22+OR+%22labour%22)+AND+(%22induction%22)+AND+(%22cervical+ripening%22)+AND+(%22misoprostol%22+OR+%22Cytotec%22)+AND+(%22randomized+trial%22)&hl=en&as_sdt=2007>

allintitle: ("labor" OR "labour") AND ("induction") AND ("cervical ripening") AND ("misoprostol" OR "Cytotec") AND ("randomized trial")

## Appendix S10. Emcare

<https://libaccess.mcmaster.ca/login?url=http://ovidsp.ovid.com/ovidweb.cgi?T=JS&NEWS=N&PAGE=main&SHAREDSEARCHID=4qHBJWlAdQjCUHtdgxuqX7NUcD2Y3JwvAwhzid6DZUvFzhb0TlYflmwErHCuQ3fRR>

| **#** | **Query** | **Results from 27 May 2025** |
| --- | --- | --- |
| 1 | labor.mp. | 66,822 |
| 2 | labour.mp. | 20,072 |
| 3 | 1 or 2 | 80,769 |
| 4 | induction.mp. | 111,786 |
| 5 | induce.mp. | 83,778 |
| 6 | 4 or 5 | 186,140 |
| 7 | 3 and 6 | 8,001 |
| 8 | uterine contraction.mp. | 507 |
| 9 | cervical.mp. | 99,901 |
| 10 | ripening.mp. | 3,313 |
| 11 | cervical ripening.mp. | 939 |
| 12 | cervical ripening agent.mp. | 27 |
| 13 | 8 or 9 or 10 or 11 or 12 | 102,547 |
| 14 | misoprostol.mp. | 5,359 |
| 15 | prostaglandin E1.mp. | 2,139 |
| 16 | PGE1.mp. | 485 |
| 17 | misodel.mp. | 15 |
| 18 | cytotec.mp. | 435 |
| 19 | 14 or 15 or 16 or 17 or 18 | 7,389 |
| 20 | 13 and 19 | 824 |
| 21 | dinoprostone.mp. | 336 |
| 22 | dinoprost.mp. | 16 |
| 23 | prostaglandin E2.mp. | 8,161 |
| 24 | PGE2.mp. | 3,847 |
| 25 | propess.mp. | 60 |
| 26 | cervidil.mp. | 72 |
| 27 | prepidil.mp. | 95 |
| 28 | prostin.mp. | 116 |
| 29 | 21 or 22 or 23 or 24 or 25 or 26 or 27 or 28 | 8,432 |
| 30 | randomized controlled trial.mp. | 420,980 |
| 31 | controlled clinical trial.mp. | 93,680 |
| 32 | randomized.ab. | 311,212 |
| 33 | placebo.ab. | 94,078 |
| 34 | drug therapy.fs. | 498,365 |
| 35 | randomly.ab. | 200,663 |
| 36 | trial.ab. | 321,881 |
| 37 | groups.ab. | 986,378 |
| 38 | 30 or 31 or 32 or 33 or 34 or 35 or 36 or 37 | 1,895,024 |
| 39 | exp animals/ not humans.sh. | 7,393,772 |
| 40 | 38 not 39 | 164,015 |
| 41 | 7 and 20 and 29 and 40 | 10 |

## Appendix S11. Cochrane

<https://www-cochranelibrary-com.libaccess.lib.mcmaster.ca/advanced-search>

((labor OR labour) AND (induction OR induce) AND (uterine contraction OR cervical OR ripening OR cervical ripening OR cervical ripening agent) AND (misoprostol OR prostaglandin E1 OR PGE1 OR misodel OR Cytotec) AND (dinoprostone OR dinoprost OR prostaglandin E2 OR PGE2 OR propess OR cervidil OR prepidil OR prostin) AND (randomized controlled trial OR controlled clinical trial OR randomized OR randomised OR placebo OR drug therapy OR trial OR groups)) NOT (animals OR humans)

# **Definition of Outcomes**

## Appendix S12. Definitions of outcomes

| **Outcome** | **Definition** |
| --- | --- |
| Critical Outcomes | |
| Cesarean births | Delivery of the baby through a surgical incision in the mother's abdomen and uterus. |
| Uterine hyperstimulation | ≥ 5 contractions in 10 minutes, or single contractions lasting ≥ 2 minutes. |
| 5-minute Apgar scores <7 | A score less than 7 at 5 minutes after birth indicating potential distress. |
| Neonatal intensive care unit (NICU) admissions | Admission of the newborn to the NICU for specialized care. |
| Oxytocin augmentation | Use of oxytocin to stimulate uterine contractions. |
| Vaginal birth within 24 hours | Achieving vaginal delivery within 24 hours of induction. |
| Induction-to-birth intervals | Time from the start of induction to the birth of the baby. |
| Important Outcomes | |
| Vaginal birth within 48 hours | Achieving vaginal delivery within 48 hours of induction. |
| Instrumental births | Assisted vaginal delivery using instruments such as forceps or vacuum. |
| Postpartum haemorrhage | Blood loss ≥ 500 ml for vaginal births or ≥ 1,000 ml for cesarean births. |
| Non-reassuring fetal heart tracings | Abnormal fetal heart rate tracings on electronic monitoring. |
| Need for tocolysis | Medical intervention to suppress premature labor. |
| Maternal adverse effects | Nausea, vomiting, diarrhea, shivering, and fever. |
| 1-minute Apgar score <7 | A score less than 7 at 1 minute after birth indicating potential distress. |
| Uterine tachysystole | ≥ 5 contractions per 10 minutes. |
| Uterine hypertonus | Single contraction ≥ 2 minutes. |
| Meconium-stained amniotic fluid | Presence of fetal stool in the amniotic fluid, indicating potential fetal distress. |
| Use of analgesia | Use of pain relief methods such as epidural and/or opioid. |

# **Excluded studies**

## Appendix S13. Table of excluded full-text articles

| **Year** | **Author** | **Reason for exclusion** | **Comment** |
| --- | --- | --- | --- |
| 1970 | Shaheen | Wrong route of administration | Compared sublingual misoprostol with vaginal dinoprostone. |
| 1997 | Arias | Conference abstract | No information available. |
| 1997 | Buser | Wrong route of administration | Compared intravaginally administered misoprostol with intracervically administered dinoprostone gel. |
| 1995 | Steytler | Full text not available | No information available. |
| 2000 | El-Din | Wrong route of administration | Compared vaginally administered misoprostol with prostaglandin E2 tablet or gel. |
| 2000 | Katz | Wrong study design | Open-label evaluation. |
| 2001 | Gherman | Wrong patient population | Patients presenting with medical or obstetric indications for labor induction whose Bishop's score was $\geq$6 were included. |
| 2002 | Barrilleaux | Wrong intervention | Compared oral misoprostol to supracervical Foley catheter and oral misoprostol with the use of a supracervical. |
| 2003 | Moodley | Wrong patient population | Patients who had a previous CS and those with a malpresentation, a non-reassuring electronic fetal heart rate recording, a Bishop's score $\geq$ 6, and partity $\geq$ 5 were excluded. |
| 2003 | Ramsey | Wrong route of administration | Compared vaginally administered misoprostol, intracervically administered dinoprostone, and vaginal insert of dinoprostone. |
| 2006 | Gupta | Wrong route of administration | Compared vaginally administered misoprostol with intracervical dinoprostone gel. |
| 2006 | Saleem | Full text not available | No information available. |
| 2009 | Nagpal | Wrong patient population  Wrong route of administration | Patients between 37 and 42 weeks’ gestation presenting with PROM at term and a Bishop score of $\leq$5.  Compared oral misoprostol with intracervical prostaglandin E2 gel. |
| 2011 | Not available | Clinical trial registration | No information available. |
| 2015 | Jha | Wrong route of administration | Compared sublingual misoprostol with intracervical dinoprostone gel. |
| 2016 | Not available | Clinical trial registration | No information available. |
| 2017 | Ayachi | Wrong route of administration | Compared vaginal misoprostol with vaginal dinoprostone insert. |
| 2018 | Not available | Full text not available | No information available. |
| 2019 | Qazi | Wrong route of administration | Compared vaginally administered misoprostol with prostaglandin E2 gel. |
| 2021 | Not available | Full text not available | No information available. |
| 2021 | Wang | Manuscript retracted |  |
| 2022 | Druenne | Wrong study design  Wrong route of administration | Prospective monocentric study that compared intravaginal dinoprostone, oral misoprostol, and double balloon catheter. |
| 2025 | Gautam | Wrong route of administration | Compared intravaginally administered misoprostol and dinoprostone gel. |
| 2025 | Mancarella | Wrong study design | Retrospective analysis comparing oral misoprostol and dinoprostone vaginal gel in patients who required a second stimulation after having already being given dinoprostone vaginal insert. |
| 2025 | Unni | Wrong route of administration | Compared vaginal misoprostol to vaginal insert dinoprostone. |

# **Risk of Bias Assessments**

## Appendix S14. Hofmeyr 2001

| **Ref or Label** | Titrated oral misoprostol solution for induction of labour: a multi-centre, randomised trial. | **Aim** | Assignment to intervention (the 'intention-to-treat' effect). | **Assessors** | TY and RA |
| --- | --- | --- | --- | --- | --- |
| **Experimental** | Oral Misoprostol | **Comparator** | Vaginal Dinoprostone | **Source** | Journal article |
| **Outcome** | Primary outcome: Vaginal delivery not achieved <24h  Secondary outcomes:  oxytocin augmentation, mode of delivery. | **Results** | Vaginal delivery within 24 hours was 62% in the oral misoprostol group and 64% in the vaginal dinoprostone group. The cesarean birth rates were 16% and 20%, respectively. | **Weight** | 1 |

| **Domain** | **Signalling question** | **Response** | **Comments** |
| --- | --- | --- | --- |
| **Bias arising from the randomization process** | 1.1 Was the allocation sequence random? | Y | The study used a computer-generated randomization sequence, a robust and standard method ensuring unpredictability. Allocation was concealed using sealed, opaque, sequentially numbered envelopes, preventing foreknowledge of assignments. |
|  | 1.2 Was the allocation sequence concealed until participants were enrolled and assigned to interventions? | Y |  |
|  | 1.3 Did baseline differences between intervention groups suggest a problem with the randomization process? | N | Baseline characteristics were well balanced, indicating successful randomization. |
|  | **Risk of bias judgement** | **Low** | The randomization process was methodologically sound, with both random sequence generation and allocation concealment clearly described and implemented. The absence of baseline imbalances further supports a low risk of bias. |
| **Bias due to deviations from intended interventions** | 2.1.Were participants aware of their assigned intervention during the trial? | Y | The study was not blinded. Participants likely knew whether they received oral or vaginal misoprostol or dinoprostone. Care providers were also not blinded, which could influence care delivery. |
|  | 2.2.Were carers and people delivering the interventions aware of participants' assigned intervention during the trial? | Y |  |
|  | 2.3. If Y/PY/NI to 2.1 or 2.2: Were there deviations from the intended intervention that arose because of the experimental context? | N | There is no indication that deviations occurred due to the open-label design. The protocol was followed consistently. |
|  | 2.4 If Y/PY to 2.3: Were these deviations likely to have affected the outcome? |  |  |
|  | 2.5. If Y/PY/NI to 2.4: Were these deviations from intended intervention balanced between groups? |  |  |
|  | 2.6 Was an appropriate analysis used to estimate the effect of assignment to intervention? | Y | The study used intention-to-treat analysis, preserving the benefits of randomization |
|  | 2.7 If N/PN/NI to 2.6: Was there potential for a substantial impact (on the result) of the failure to analyse participants in the group to which they were randomized? |  |  |
|  | **Risk of bias judgement** | **Some concerns** | The lack of blinding introduces the potential for performance bias, as both participants and caregivers may have altered behaviour based on group assignment. Although no deviations were reported and the analysis was appropriate, the unblinded design leads to some concerns. |
| **Bias due to missing outcome data** | 3.1 Were data for this outcome available for all, or nearly all, participants randomized? | Y | The study analysed 250 out of 300 randomized participants. The 50 exclusions were due to predefined and documented reasons (e.g., protocol violations, clerical errors). |
|  | 3.2 If N/PN/NI to 3.1: Is there evidence that result was not biased by missing outcome data? | Y | Missing data were balanced and unrelated to outcomes, and all participants were accounted for. |
|  | 3.3 If N/PN to 3.2: Could missingness in the outcome depend on its true value? |  |  |
|  | 3.4 If Y/PY/NI to 3.3: Is it likely that missingness in the outcome depended on its true value? |  |  |
|  | **Risk of bias judgement** | **Low** | The completeness of outcome data and the lack of differential loss to follow-up support the reliability of the results. There is no indication that missing data introduced bias. |
| **Bias in measurement of the outcome** | 4.1 Was the method of measuring the outcome inappropriate? | N | Outcomes were measured using standard clinical definitions and hospital records. |
|  | 4.2 Could measurement or ascertainment of the outcome have differed between intervention groups? | Y | Because the study was not blinded, outcome assessment could have been influenced by knowledge of group assignment. |
|  | 4.3 Were outcome assessors aware of the intervention received by study participants? | Y | There is no indication that outcome assessors were blinded, increasing the risk of detection bias. |
|  | 4.4 If Y/PY/NI to 4.3: Could assessment of the outcome have been influenced by knowledge of intervention received? | Y | When assessors are aware of the treatment groups, their expectations may impact their evaluations. For example, fetal distress was a subjective outcome based on CTG interpretation, which could be influenced by knowledge of the intervention. |
|  | 4.5 If Y/PY/NI to 4.4: Is it likely that assessment of the outcome was influenced by knowledge of intervention received? | N |  |
|  | **Risk of bias judgement** | **Some concerns** | Although many outcomes were objective (e.g., mode of delivery), others involved clinical judgment (e.g., decision to perform cesarean section). The lack of blinding among outcome assessors introduces the potential for bias in outcome measurement. |
| **Bias in selection of the reported result** | 5.1 Were the data that produced this result analysed in accordance with a pre-specified analysis plan that was finalized before unblinded outcome data were available for analysis? | PY | The outcomes reported match those listed in the methods section, suggesting adherence to a plan. However, the absence of a publicly available protocol or trial registration means this cannot be confirmed with certainty. |
|  | 5.2 ... multiple eligible outcome measurements (e.g. scales, definitions, time points) within the outcome domain? | Y | The study reported on several outcomes (e.g., delivery time, cesarean indications, fetal distress), and it’s unclear if these were pre-specified or selectively reported. |
|  | 5.3 ... multiple eligible analyses of the data? | Y | The study could have conducted multiple subgroup or post hoc analyses, but this is not addressed in the methods or results. |
|  | **Risk of bias judgement** | **Some concerns** | Although the reported outcomes align with those described in the methods, the lack of a pre-registered protocol or analysis plan introduces uncertainty. The presence of multiple outcome measures and the potential for unreported analyses raise the possibility of selective reporting or analysis. |
| **Overall bias** | **Risk of bias judgement** | **Some concerns** | While the study demonstrates strong methodology in randomization, outcome completeness, and reporting, the lack of blinding introduces some concerns in both the deviation from intended interventions and outcome measurement domains. These concerns do not invalidate the results but suggest that the findings should be interpreted with caution, particularly for outcomes that could be influenced by knowledge of treatment allocation. |

## Appendix S15. le Roux 2002

| **Ref or Label** | Oral and Vaginal Misoprostol Compared With Dinoprostone for Induction of Labor | **Aim** | Assignment to intervention (the 'intention-to-treat' effect) | **Assessors** | TY and RA |
| --- | --- | --- | --- | --- | --- |
| **Experimental** | Oral Misoprostol | **Comparator** | Vaginal Dinoprostone | **Source** | Journal article |
| **Outcome** | Rate of vaginal delivery within 24 hours, median induction-to-delivery time, and cesarean section rates. | **Results** | Rate of vaginal delivery within 24 hours (39% for misoprostol, and 54% for dinoprostone), median induction-to-delivery time (22.6 hours, and 14.8 hours, respectively), and cesarean births (approx. 33% in both) | **Weight** | 1 |

| **Domain** | **Signalling question** | **Response** | **Comments** |
| --- | --- | --- | --- |
| **Bias arising from the randomization process** | 1.1 Was the allocation sequence random? | Y | The study used computer-generated randomization, which is a robust method for ensuring random allocation.  Sealed opaque envelopes were used, opened only after participant enrolment. This method is widely accepted for maintaining allocation concealment. |
|  | 1.2 Was the allocation sequence concealed until participants were enrolled and assigned to interventions? | Y |  |
|  | 1.3 Did baseline differences between intervention groups suggest a problem with the randomization process? | N | Demographic characteristics and baseline measures, such as age, gravidity, parity, and Bishop scores, were well balanced across the groups. |
|  | **Risk of bias judgement** | **Low** | The randomization process was clearly described and appropriately implemented. There were no signs of allocation bias or baseline imbalances, supporting a low risk of bias. |
| **Bias due to deviations from intended interventions** | 2.1.Were participants aware of their assigned intervention during the trial? | Y | The study was not blinded; participants knew their assigned treatment.  Medical staff administering the interventions were also not blinded. |
|  | 2.2.Were carers and people delivering the interventions aware of participants' assigned intervention during the trial? | Y |  |
|  | 2.3. If Y/PY/NI to 2.1 or 2.2: Were there deviations from the intended intervention that arose because of the experimental context? | N | The study protocol was followed consistently. There is no evidence of deviations due to the open-label design. |
|  | 2.4 If Y/PY to 2.3: Were these deviations likely to have affected the outcome? |  |  |
|  | 2.5. If Y/PY/NI to 2.4: Were these deviations from intended intervention balanced between groups? |  |  |
|  | 2.6 Was an appropriate analysis used to estimate the effect of assignment to intervention? | Y | The analysis was conducted on the randomized groups using intention-to-treat principles. Although 93 participants were excluded due to protocol violations, the reasons were documented and mostly administrative (e.g., clerical errors, ineligibility). |
|  | 2.7 If N/PN/NI to 2.6: Was there potential for a substantial impact (on the result) of the failure to analyse participants in the group to which they were randomized? |  |  |
|  | **Risk of bias judgement** | **Some concerns** | The lack of blinding introduces the potential for performance bias, especially in a labor ward setting where clinical decisions could be influenced by knowledge of treatment. However, no deviations were reported, and the analysis was appropriate. |
| **Bias due to missing outcome data** | 3.1 Were data for this outcome available for all, or nearly all, participants randomized? | Y | Of the 573 women randomized, 480 were included in the final analysis. The 93 exclusions were due to predefined and documented reasons (e.g., clerical errors, ineligibility, withdrawal). |
|  | 3.2 If N/PN/NI to 3.1: Is there evidence that result was not biased by missing outcome data? | Y | The exclusions were not related to outcomes and were evenly distributed across groups. |
|  | 3.3 If N/PN to 3.2: Could missingness in the outcome depend on its true value? |  |  |
|  | 3.4 If Y/PY/NI to 3.3: Is it likely that missingness in the outcome depended on its true value? |  |  |
|  | **Risk of bias judgement** | **Low** | The missing data were well explained and unlikely to introduce bias. |
| **Bias in measurement of the outcome** | 4.1 Was the method of measuring the outcome inappropriate? | N | Outcomes such as vaginal delivery within 24 hours, cesarean rates, and fetal distress were standard and well defined. |
|  | 4.2 Could measurement or ascertainment of the outcome have differed between intervention groups? | Y | Outcome assessors were not blinded, and some outcomes (e.g., fetal distress) involve clinical judgment. |
|  | 4.3 Were outcome assessors aware of the intervention received by study participants? | Y | Outcome assessors were aware of group assignments, as the study was not blinded. This could introduce potential bias in subjective outcomes like fetal monitoring interpretation. |
|  | 4.4 If Y/PY/NI to 4.3: Could assessment of the outcome have been influenced by knowledge of intervention received? | PY | Fetal distress was determined based on CTG interpretation, which is subjective and could be influenced by knowledge of the intervention. Given the subjective nature of CTG interpretation and the lack of blinding, there is a plausible risk of detection bias. |
|  | 4.5 If Y/PY/NI to 4.4: Is it likely that assessment of the outcome was influenced by knowledge of intervention received? | PY |  |
|  | **Risk of bias judgement** | **Some concerns** | The lack of blinding in outcome assessment, particularly for fetal distress, introduces the potential for detection bias |
| **Bias in selection of the reported result** | 5.1 Were the data that produced this result analysed in accordance with a pre-specified analysis plan that was finalized before unblinded outcome data were available for analysis? | PY | Outcomes reported match those listed in the methods, but no protocol or registration is cited. |
|  | 5.2 ... multiple eligible outcome measurements (e.g. scales, definitions, time points) within the outcome domain? | Y | The study reported several outcomes (e.g., delivery time, cesarean indications, fetal distress), and it’s unclear if all were pre-specified. |
|  | 5.3 ... multiple eligible analyses of the data? | N |  |
|  | **Risk of bias judgement** | **Some concerns** | The absence of a pre-specified analysis plan and the presence of multiple outcome measures and potential analyses raise the possibility of selective reporting. |
| **Overall bias** | **Risk of bias judgement** | **Some concerns** | While the study was well randomized and had minimal missing data, the lack of blinding in both intervention delivery and outcome assessment, combined with uncertainty about pre-specification of outcomes, introduces some concerns. These do not invalidate the findings but suggest that results should be interpreted with caution. |

## Appendix S16. Dällenbach 2003

| **Ref or Label** | Oral Misoprostol or Vaginal Dinoprostone for Labor Induction: A Randomized Controlled Trial | **Aim** | Assignment to intervention (the 'intention-to-treat' effect) | **Assessors** | TY and RA |
| --- | --- | --- | --- | --- | --- |
| **Experimental** | Oral Misoprostol | **Comparator** | Vaginal Dinoprostone | **Source** | Journal article |
| **Outcome** | Vaginal delivery within 24 hours (primary).  Cesarean section rates, time to delivery, uterine abnormalities, and neonatal outcomes (secondary). | **Results** | No significant differences in vaginal delivery within 24 hours or cesarean births. Misoprostol had a longer time to delivery and more thick meconium but no differences in Apgar scores or NICU admissions. Maternal side effects were higher with misoprostol. | **Weight** | 1 |

| **Domain** | **Signalling question** | **Response** | **Comments** |
| --- | --- | --- | --- |
| **Bias arising from the randomization process** | 1.1 Was the allocation sequence random? | Y | The study used computer-generated randomization with randomly permuted blocks.  Allocation was concealed using sealed, consecutively numbered opaque envelopes. |
|  | 1.2 Was the allocation sequence concealed until participants were enrolled and assigned to interventions? | Y |  |
|  | 1.3 Did baseline differences between intervention groups suggest a problem with the randomization process? | N | Baseline characteristics were similar between groups, except for slightly higher preinduction Bishop scores in the dinoprostone group. Adjustments were made for this in the analysis. |
|  | **Risk of bias judgement** | **Low** | The randomization process was clearly described and appropriately implemented. Minor baseline differences were accounted for in the analysis. |
| **Bias due to deviations from intended interventions** | 2.1.Were participants aware of their assigned intervention during the trial? | Y | The study was not blinded, meaning both participants and clinicians were aware of the intervention.  Clinicians delivering the interventions were aware of the assigned treatment. |
|  | 2.2.Were carers and people delivering the interventions aware of participants' assigned intervention during the trial? | Y |  |
|  | 2.3. If Y/PY/NI to 2.1 or 2.2: Were there deviations from the intended intervention that arose because of the experimental context? | N | The interventions were followed as per protocol with minimal deviation, indicating that the treatment regimens were strictly adhered to. |
|  | 2.4 If Y/PY to 2.3: Were these deviations likely to have affected the outcome? |  |  |
|  | 2.5. If Y/PY/NI to 2.4: Were these deviations from intended intervention balanced between groups? |  |  |
|  | 2.6 Was an appropriate analysis used to estimate the effect of assignment to intervention? | Y | Intention-to-treat analysis was used, preserving the randomization and including all participants. |
|  | 2.7 If N/PN/NI to 2.6: Was there potential for a substantial impact (on the result) of the failure to analyse participants in the group to which they were randomized? |  |  |
|  | **Risk of bias judgement** | **Some concerns** | The lack of blinding introduces the potential for performance bias, particularly in decisions about oxytocin use or cesarean delivery. However, no deviations were reported, and the analysis was appropriate. |
| **Bias due to missing outcome data** | 3.1 Were data for this outcome available for all, or nearly all, participants randomized? | Y | 200 of 202 randomized women were included in the analysis; the two exclusions were protocol violations. |
|  | 3.2 If N/PN/NI to 3.1: Is there evidence that result was not biased by missing outcome data? | Y | The exclusions were unrelated to outcomes and balanced across groups. |
|  | 3.3 If N/PN to 3.2: Could missingness in the outcome depend on its true value? |  |  |
|  | 3.4 If Y/PY/NI to 3.3: Is it likely that missingness in the outcome depended on its true value? |  |  |
|  | **Risk of bias judgement** | **Low** | The small number of exclusions was well documented and unlikely to introduce bias. |
| **Bias in measurement of the outcome** | 4.1 Was the method of measuring the outcome inappropriate? | N | The outcomes were measured using standard clinical methods, such as vaginal delivery rates, cesarean sections, and uterine contractility abnormalities. |
|  | 4.2 Could measurement or ascertainment of the outcome have differed between intervention groups? | Y | The study was not blinded, and some outcomes (e.g., decision to perform cesarean or use oxytocin) could be influenced by knowledge of treatment. |
|  | 4.3 Were outcome assessors aware of the intervention received by study participants? | Y | Clinical staff were not blinded, but cardiotocograph traces were reviewed by a blinded assessor. |
|  | 4.4 If Y/PY/NI to 4.3: Could assessment of the outcome have been influenced by knowledge of intervention received? | Y | \| The knowledge of the intervention could influence the assessment of some outcomes, especially subjective ones like fetal distress or hyperstimulation. The independent review of fetal heart rate data by blinded observers mitigates this risk.  While the outcome assessors were aware of the intervention, the independent blinded review of key outcomes (e.g., cardiotocograms) suggests it is unlikely that the overall assessment was influenced. \| \| --- \| |
|  | 4.5 If Y/PY/NI to 4.4: Is it likely that assessment of the outcome was influenced by knowledge of intervention received? | N |  |
|  | **Risk of bias judgement** | **Some concerns** | Although objective outcomes were used and some assessments were blinded, the lack of blinding in clinical decision-making introduces potential detection bias. Therefore, this domain is rated as having some concerns. |
| **Bias in selection of the reported result** | 5.1 Were the data that produced this result analysed in accordance with a pre-specified analysis plan that was finalized before unblinded outcome data were available for analysis? | PY | The study followed a pre-specified analysis plan, and all outcomes were reported as planned. |
|  | 5.2 ... multiple eligible outcome measurements (e.g. scales, definitions, time points) within the outcome domain? | Y | The study reported multiple outcomes, including vaginal delivery rates, cesarean sections, and maternal and neonatal complications, providing a comprehensive evaluation of the interventions. |
|  | 5.3 ... multiple eligible analyses of the data? | Y | The study used various statistical methods, including relative risk, survival analysis, and Cox models, to analyse different outcomes and subgroups, which ensures thorough analysis of the data. |
|  | **Risk of bias judgement** | **Low** | The study adhered to a pre-specified analysis plan, with all planned outcomes reported. This reduces the risk of selective reporting, ensuring that the findings were not manipulated to favour the results of any specific intervention. |
| **Overall bias** | **Risk of bias judgement** | **Low** | Although the study was not blinded, it employed robust randomization, allocation concealment, intention-to-treat analysis, and blinded assessment of key outcomes (e.g., cardiotocograph traces). The comprehensive and transparent reporting of outcomes, along with appropriate statistical methods, supports a low overall risk of bias. |

## Appendix S17. Matonhodze 2003

| **Ref or Label** | Labour induction at term - a randomised trial comparing Foley catheter plus titrated oral misoprostol solution, titrated oral misoprostol solution alone, and dinoprostone | **Aim** | Assignment to intervention (the 'intention-to-treat' effect) | **Assessors** | TY and RA |
| --- | --- | --- | --- | --- | --- |
| **Experimental** | Oral Misoprostol | **Comparator** | Vaginal Dinoprostone | **Source** | Journal article |
| **Outcome** | Failure to deliver vaginally within 24 hours, additional measures for induction or augmentation of labour, analgesia, and maternal and fetal complications. | **Results** | Failure to deliver vaginally within 24 hours was similar in both groups (PGE1 70/176 v. PGE2 70/177). | **Weight** | 1 |

| **Domain** | **Signalling question** | **Response** | **Comments** |
| --- | --- | --- | --- |
| **Bias arising from the randomization process** | 1.1 Was the allocation sequence random? | Y | The study used a computer-generated random sequence with sealed, opaque, and numbered envelopes to ensure proper allocation.  Allocation was concealed using sealed opaque envelopes, preventing foreknowledge of assignments. |
|  | 1.2 Was the allocation sequence concealed until participants were enrolled and assigned to interventions? | Y |  |
|  | 1.3 Did baseline differences between intervention groups suggest a problem with the randomization process? | N | Groups were comparable across baseline characteristics, with no significant imbalances. |
|  | **Risk of bias judgement** | **Low** | The randomization process was clearly described and appropriately implemented. There were no signs of allocation bias or significant baseline imbalances. |
| **Bias due to deviations from intended interventions** | 2.1.Were participants aware of their assigned intervention during the trial? | Y | Both participants and care providers were aware of the intervention due to lack of blinding.  Clinical staff knew the intervention assignments, potentially influencing their behaviour. |
|  | 2.2.Were carers and people delivering the interventions aware of participants' assigned intervention during the trial? | Y |  |
|  | 2.3. If Y/PY/NI to 2.1 or 2.2: Were there deviations from the intended intervention that arose because of the experimental context? | N | Treatment protocols were followed as per study design, with no evidence of deviations arising from the trial context. |
|  | 2.4 If Y/PY to 2.3: Were these deviations likely to have affected the outcome? |  |  |
|  | 2.5. If Y/PY/NI to 2.4: Were these deviations from intended intervention balanced between groups? |  |  |
|  | 2.6 Was an appropriate analysis used to estimate the effect of assignment to intervention? | Y | Intention-to-treat analysis was used, which mitigates potential bias from protocol deviations. |
|  | 2.7 If N/PN/NI to 2.6: Was there potential for a substantial impact (on the result) of the failure to analyse participants in the group to which they were randomized? |  |  |
|  | **Risk of bias judgement** | **Some concerns** | While the lack of blinding introduces some concerns, adherence to protocol and robust analysis methods reduce overall bias risk. |
| **Bias due to missing outcome data** | 3.1 Were data for this outcome available for all, or nearly all, participants randomized? | Y | Nearly all participants were analysed. Missing data were minimal and unrelated to study outcomes. |
|  | 3.2 If N/PN/NI to 3.1: Is there evidence that result was not biased by missing outcome data? | Y | The small number of missing data points was balanced and unlikely to affect the results. |
|  | 3.3 If N/PN to 3.2: Could missingness in the outcome depend on its true value? |  |  |
|  | 3.4 If Y/PY/NI to 3.3: Is it likely that missingness in the outcome depended on its true value? |  |  |
|  | **Risk of bias judgement** | **Low** | The completeness of outcome data and the lack of differential loss to follow-up support a low risk of bias. |
| **Bias in measurement of the outcome** | 4.1 Was the method of measuring the outcome inappropriate? | N | Standard clinical methods were used for outcome measurement (e.g., delivery rates, uterine contractions). |
|  | 4.2 Could measurement or ascertainment of the outcome have differed between intervention groups? | Y | The study was not blinded, and some outcomes (e.g., decision to use analgesia or perform cesarean) could be influenced by knowledge of treatment. |
|  | 4.3 Were outcome assessors aware of the intervention received by study participants? | Y | Clinical staff were not blinded, but cardiotocograph traces were reviewed by a blinded assessor. |
|  | 4.4 If Y/PY/NI to 4.3: Could assessment of the outcome have been influenced by knowledge of intervention received? | Y | Knowledge of the intervention could influence the assessment of subjective outcomes like uterine hyperstimulation or fetal distress. However, blinded review of cardiotocogram (CTG) data mitigated this risk for critical measures. |
|  | 4.5 If Y/PY/NI to 4.4: Is it likely that assessment of the outcome was influenced by knowledge of intervention received? | N |  |
|  | **Risk of bias judgement** | **Some concerns** | Although objective outcomes were used and some assessments were blinded, the lack of blinding in clinical decision-making introduces potential detection bias. |
| **Bias in selection of the reported result** | 5.1 Were the data that produced this result analysed in accordance with a pre-specified analysis plan that was finalized before unblinded outcome data were available for analysis? | Y | A pre-specified plan was followed, and all relevant outcomes were reported as intended. |
|  | 5.2 ... multiple eligible outcome measurements (e.g. scales, definitions, time points) within the outcome domain? | Y | The study reported a wide range of outcomes across maternal, fetal, and labor process domains. These included delivery within 24 hours, augmentation methods, uterine activity patterns, analgesia use, and neonatal outcomes. All were reported transparently and consistently, with no evidence of selective emphasis. |
|  | 5.3 ... multiple eligible analyses of the data? | Y | The study used relative risks with 95% confidence intervals, subgroup analyses (e.g., by cervical status), and intention-to-treat analysis. These were described in the methods and applied consistently. There’s no indication of post hoc data dredging or selective analysis. |
|  | **Risk of bias judgement** | **Low** | The study followed a clear and pre-specified analysis plan, reported all outcomes comprehensively, and used appropriate statistical methods. There is no evidence of selective reporting or analysis manipulation. |
| **Overall bias** | **Risk of bias judgement** | **Low** | While the study was not blinded, this limitation was mitigated by the use of objective outcomes and blinded assessment of key safety data. There is no evidence of deviations from protocol or selective reporting. Therefore, the study’s findings are considered reliable, and the overall risk of bias is low. |

## Appendix S18. Shetty 2004

| **Ref or Label** | A randomised comparison of oral misoprostol and vaginal prostaglandin E2 tablets in labour induction at term | **Aim** | Assignment to intervention (the 'intention-to-treat' effect) | **Assessors** | TY and RA |
| --- | --- | --- | --- | --- | --- |
| **Experimental** | Oral Misoprostol (PGE1) | **Comparator** | Vaginal Dinoprostone (PGE2) | **Source** | Journal article |
| **Outcome** | The number delivering vaginally within 24 hours of the induction. | **Results** | 50.7% in the misoprostol group and 54.8% in the PGE2 group delivered vaginally within 24 hours of the induction. | **Weight** | 1 |

| **Domain** | **Signalling question** | **Response** | **Comments** |
| --- | --- | --- | --- |
| **Bias arising from the randomization process** | 1.1 Was the allocation sequence random? | Y | The study used a computer-generated randomization table, and allocation was concealed using opaque envelopes.  Allocation concealment was ensured via sealed, sequentially numbered, opaque envelopes, preventing foreknowledge of assignments. |
|  | 1.2 Was the allocation sequence concealed until participants were enrolled and assigned to interventions? | Y |  |
|  | 1.3 Did baseline differences between intervention groups suggest a problem with the randomization process? | N | Groups were comparable in terms of demographic characteristics and clinical indications for induction, with no significant imbalances. |
|  | **Risk of bias judgement** | **Low** | Robust randomization and allocation concealment ensured fair group assignments, minimizing selection bias. |
| **Bias due to deviations from intended interventions** | 2.1.Were participants aware of their assigned intervention during the trial? | Y | The study was non-blinded, meaning participants and caregivers knew the intervention assigned, introducing the potential for bias due to performance effects.  Care providers were aware of the interventions, potentially influencing decisions on management such as oxytocin augmentation. |
|  | 2.2.Were carers and people delivering the interventions aware of participants' assigned intervention during the trial? | Y |  |
|  | 2.3. If Y/PY/NI to 2.1 or 2.2: Were there deviations from the intended intervention that arose because of the experimental context? | N | Interventions were delivered as per protocol. |
|  | 2.4 If Y/PY to 2.3: Were these deviations likely to have affected the outcome? |  |  |
|  | 2.5. If Y/PY/NI to 2.4: Were these deviations from intended intervention balanced between groups? |  |  |
|  | 2.6 Was an appropriate analysis used to estimate the effect of assignment to intervention? | Y | Intention-to-treat analysis was employed, preserving the benefits of randomization and accounting for all participants in their original groups. |
|  | 2.7 If N/PN/NI to 2.6: Was there potential for a substantial impact (on the result) of the failure to analyse participants in the group to which they were randomized? |  |  |
|  | **Risk of bias judgement** | **Some concerns** | The lack of blinding introduces the potential for performance bias, especially in subjective decisions like oxytocin use or timing of cesarean. However, the protocol was followed, and analysis was appropriate. |
| **Bias due to missing outcome data** | 3.1 Were data for this outcome available for all, or nearly all, participants randomized? | Y | All 200 randomized women were included in the analysis. |
|  | 3.2 If N/PN/NI to 3.1: Is there evidence that result was not biased by missing outcome data? | Y | There were no missing data reported. |
|  | 3.3 If N/PN to 3.2: Could missingness in the outcome depend on its true value? |  |  |
|  | 3.4 If Y/PY/NI to 3.3: Is it likely that missingness in the outcome depended on its true value? |  |  |
|  | **Risk of bias judgement** | **Low** | Complete outcome data eliminates risk of bias due to missing data. |
| **Bias in measurement of the outcome** | 4.1 Was the method of measuring the outcome inappropriate? | N | Outcomes were measured using standard clinical methods (e.g., vaginal delivery within 24 hours, uterine hyperstimulation). |
|  | 4.2 Could measurement or ascertainment of the outcome have differed between intervention groups? | Y | Non-blinded assessors may have introduced bias in subjective outcomes (e.g., uterine hyperstimulation), but objective outcomes like delivery timing were less prone to bias. |
|  | 4.3 Were outcome assessors aware of the intervention received by study participants? | Y | Assessors were not blinded to interventions, which could influence subjective evaluations. |
|  | 4.4 If Y/PY/NI to 4.3: Could assessment of the outcome have been influenced by knowledge of intervention received? | Y | Subjective measures like uterine hyperstimulation might have been influenced by intervention knowledge. However, key objective outcomes were not affected.  The reliance on objective measures such as delivery timing mitigates the likelihood of significant bias. |
|  | 4.5 If Y/PY/NI to 4.4: Is it likely that assessment of the outcome was influenced by knowledge of intervention received? | N |  |
|  | **Risk of bias judgement** | **Some concerns** | Although many outcomes were objective, the lack of blinding in outcome assessment introduces the potential for detection bias, especially for labor management decisions. |
| **Bias in selection of the reported result** | 5.1 Were the data that produced this result analysed in accordance with a pre-specified analysis plan that was finalized before unblinded outcome data were available for analysis? | Y | The study followed a pre-specified analysis plan, reporting outcomes as planned. |
|  | 5.2 ... multiple eligible outcome measurements (e.g. scales, definitions, time points) within the outcome domain? | Y | Multiple outcomes were reported (e.g., delivery within 24 hours, oxytocin use, hyperstimulation, neonatal outcomes), but all were transparently presented. |
|  | 5.3 ... multiple eligible analyses of the data? | Y | The study used relative risks, confidence intervals, and subgroup analyses, but these were appropriate and not selectively emphasized. |
|  | **Risk of bias judgement** | **Low** | Comprehensive reporting and adherence to the analysis plan suggest minimal risk of selective reporting bias. |
| **Overall bias** | **Risk of bias judgement** | **Some concerns** | The study was well randomized, had complete outcome data, and reported results transparently. However, the lack of blinding for participants and clinicians introduces some concerns in both the deviation from intended interventions and measurement of outcomes domains. |

## Appendix S19. Dodd 2006

| **Ref or Label** | Oral misoprostol for induction of labour at term: randomised controlled trial | **Aim** | Assignment to intervention (the 'intention-to-treat' effect) | **Assessors** | TY and RA |
| --- | --- | --- | --- | --- | --- |
| **Experimental** | Oral Misoprostol | **Comparator** | Vaginal Dinoprostone | **Source** | Journal article |
| **Outcome** | Vaginal birth within 24 hours; uterine hyperstimulation with associated changes in fetal heart rate; cesarean section (all); and cesarean section for fetal distress. | **Results** | No significant differences between the two treatment groups in the primary outcomes: vaginal birth not achieved in 24 hours (misoprostol 168/365 (46.0%) vs. dinoprostone 155/376 (41.2%). | **Weight** | 1 |

| **Domain** | **Signalling question** | **Response** | **Comments** |
| --- | --- | --- | --- |
| **Bias arising from the randomization process** | 1.1 Was the allocation sequence random? | Y | Randomization was performed using a computer-generated sequence with variable blocks, stratified by parity and centre.  Allocation was concealed using sealed, sequentially numbered treatment packs, ensuring allocation concealment. |
|  | 1.2 Was the allocation sequence concealed until participants were enrolled and assigned to interventions? | Y |  |
|  | 1.3 Did baseline differences between intervention groups suggest a problem with the randomization process? | N | Baseline characteristics were comparable between groups, except for initial Bishop scores, which were adjusted for in the analysis. |
|  | **Risk of bias judgement** | **Low** | Proper randomization and allocation concealment minimize selection bias, and adjustments for imbalances reduce the risk of confounding. |
| **Bias due to deviations from intended interventions** | 2.1.Were participants aware of their assigned intervention during the trial? | N | Participants were blinded to their treatment group due to the use of identical treatment packs and solutions.  Care providers were also blinded to the treatment assignment through identical treatment packs. |
|  | 2.2.Were carers and people delivering the interventions aware of participants' assigned intervention during the trial? | Y |  |
|  | 2.3. If Y/PY/NI to 2.1 or 2.2: Were there deviations from the intended intervention that arose because of the experimental context? | N | The study was double-blinded, meaning neither participants nor carers were aware of the assigned intervention. Additionally, there is no evidence of deviations from the intended interventions arising from trial context or other factors. The protocol was strictly followed. |
|  | 2.4 If Y/PY to 2.3: Were these deviations likely to have affected the outcome? |  |  |
|  | 2.5. If Y/PY/NI to 2.4: Were these deviations from intended intervention balanced between groups? |  |  |
|  | 2.6 Was an appropriate analysis used to estimate the effect of assignment to intervention? | Y | Intention-to-treat analysis was employed, preserving randomization and accounting for all participants as allocated. |
|  | 2.7 If N/PN/NI to 2.6: Was there potential for a substantial impact (on the result) of the failure to analyse participants in the group to which they were randomized? |  |  |
|  | **Risk of bias judgement** | **Low** | Blinding of participants and carers, along with robust analysis, minimizes bias from deviations. |
| **Bias due to missing outcome data** | 3.1 Were data for this outcome available for all, or nearly all, participants randomized? | Y | Outcome data were available for all randomized participants up to hospital discharge. |
|  | 3.2 If N/PN/NI to 3.1: Is there evidence that result was not biased by missing outcome data? | N | There was no missing data. |
|  | 3.3 If N/PN to 3.2: Could missingness in the outcome depend on its true value? |  |  |
|  | 3.4 If Y/PY/NI to 3.3: Is it likely that missingness in the outcome depended on its true value? |  |  |
|  | **Risk of bias judgement** | **Low** | Complete outcome data eliminate risks from attrition bias. |
| **Bias in measurement of the outcome** | 4.1 Was the method of measuring the outcome inappropriate? | N | Outcome measures, including vaginal delivery, cesarean sections, and uterine hyperstimulation, were defined using standard clinical criteria. |
|  | 4.2 Could measurement or ascertainment of the outcome have differed between intervention groups? | N | Measurement was consistent across groups due to blinding of the treatment group. |
|  | 4.3 Were outcome assessors aware of the intervention received by study participants? | N | Outcome assessors were blinded to the treatment groups, reducing the potential for differential assessment bias. |
|  | 4.4 If Y/PY/NI to 4.3: Could assessment of the outcome have been influenced by knowledge of intervention received? | N | Blinded outcome assessment prevents influence from knowledge of treatment.  The comprehensive blinding of assessors ensures unbiased outcome measurement. |
|  | 4.5 If Y/PY/NI to 4.4: Is it likely that assessment of the outcome was influenced by knowledge of intervention received? | N |  |
|  | **Risk of bias judgement** | **Low** | Proper blinding and objective measures ensure low risk of bias in outcome measurement. |
| **Bias in selection of the reported result** | 5.1 Were the data that produced this result analysed in accordance with a pre-specified analysis plan that was finalized before unblinded outcome data were available for analysis? | Y | The study adhered to a pre-specified analysis plan, with primary and secondary outcomes clearly defined and reported. |
|  | 5.2 ... multiple eligible outcome measurements (e.g. scales, definitions, time points) within the outcome domain? | Y | Multiple outcomes were measured and reported (e.g., vaginal birth rates, uterine hyperstimulation, neonatal outcomes), providing a comprehensive evaluation of the intervention effects. |
|  | 5.3 ... multiple eligible analyses of the data? | Y | Data were analysed using intention-to-treat principles, including appropriate statistical adjustments for baseline imbalances. |
|  | **Risk of bias judgement** | **Low** | Transparent reporting and pre-specified analysis minimize risks of selective reporting bias. |
| **Overall bias** | **Risk of bias judgement** | **Low** | This was a well-designed, double-blind, placebo-controlled randomized trial with robust randomization, allocation concealment, complete outcome data, blinded outcome assessment, and transparent reporting. There is no evidence of bias across any domain, and the study’s findings are reliable. |

## Appendix S20. Henrich 2008

| **Ref or Label** | Oral misoprostol against vaginal dinoprostone for labor induction at term: a randomized comparison | **Aim** | Assignment to intervention (the 'intention-to-treat' effect) | **Assessors** | TY and RA |
| --- | --- | --- | --- | --- | --- |
| **Experimental** | Oral Misoprostol | **Comparator** | Vaginal Dinoprostone | **Source** | Journal article |
| **Outcome** | The duration and mode of labor were investigated as primary endpoints, as well as maternal and neonatal outcomes, side effects and costs. | **Results** | In the dinoprostone group,  the median duration of labour was 17.6 hours compared to 24.1 hours in the misoprostol group. In the dinoprostone group, the rate of spontaneous births within 24 hours was higher (53.6% vs. 41.1%). | **Weight** | 1 |

| **Domain** | **Signalling question** | **Response** | **Comments** |
| --- | --- | --- | --- |
| **Bias arising from the randomization process** | 1.1 Was the allocation sequence random? | Y | Randomized, sealed opaque envelopes with random block sizes were used, ensuring proper randomization.  The use of sealed, consecutively numbered opaque envelopes ensured that allocation was concealed until participants were enrolled. |
|  | 1.2 Was the allocation sequence concealed until participants were enrolled and assigned to interventions? | Y |  |
|  | 1.3 Did baseline differences between intervention groups suggest a problem with the randomization process? | N | Baseline characteristics were balanced across groups, confirming the randomization was effective. |
|  | **Risk of bias judgement** | **Low** | The allocation sequence was random, employing sealed, opaque envelopes and random block sizes. This ensured proper randomization and concealed allocation.  Baseline characteristics were well-balanced between the intervention groups, as stated in the study, indicating that randomization was effective and unbiased. |
| **Bias due to deviations from intended interventions** | 2.1.Were participants aware of their assigned intervention during the trial? | Y | Participants and clinicians were aware of assigned interventions due to the lack of placebo control.  Clinicians were aware of interventions, as the study was not blinded. |
|  | 2.2.Were carers and people delivering the interventions aware of participants' assigned intervention during the trial? | Y |  |
|  | 2.3. If Y/PY/NI to 2.1 or 2.2: Were there deviations from the intended intervention that arose because of the experimental context? | N | The study adhered to protocols, and deviations were unlikely as interventions followed standard procedures unless clinically necessary. |
|  | 2.4 If Y/PY to 2.3: Were these deviations likely to have affected the outcome? |  |  |
|  | 2.5. If Y/PY/NI to 2.4: Were these deviations from intended intervention balanced between groups? |  |  |
|  | 2.6 Was an appropriate analysis used to estimate the effect of assignment to intervention? | Y | The study used intention-to-treat analysis, preserving randomization integrity and ensuring unbiased effect estimation. |
|  | 2.7 If N/PN/NI to 2.6: Was there potential for a substantial impact (on the result) of the failure to analyse participants in the group to which they were randomized? |  |  |
|  | **Risk of bias judgement** | **Some concerns** | The lack of blinding introduces the potential for performance bias, especially in subjective decisions like timing of oxytocin or vacuum extraction. However, the protocol was followed, and analysis was appropriate. |
| **Bias due to missing outcome data** | 3.1 Were data for this outcome available for all, or nearly all, participants randomized? | Y | Outcome data were available for all 224 randomized participants. |
|  | 3.2 If N/PN/NI to 3.1: Is there evidence that result was not biased by missing outcome data? |  |  |
|  | 3.3 If N/PN to 3.2: Could missingness in the outcome depend on its true value? |  |  |
|  | 3.4 If Y/PY/NI to 3.3: Is it likely that missingness in the outcome depended on its true value? |  |  |
|  | **Risk of bias judgement** | **Low** | Outcome data were available for all 224 randomized participants. There was no missing data. |
| **Bias in measurement of the outcome** | 4.1 Was the method of measuring the outcome inappropriate? | N | Outcomes such as vaginal delivery within 24 hours and cesarean section rates are standard, well-defined measures in obstetric research. |
|  | 4.2 Could measurement or ascertainment of the outcome have differed between intervention groups? | Y | The study was not blinded, and some outcomes (e.g., decision to use vacuum extraction or oxytocin) could be influenced by knowledge of treatment. |
|  | 4.3 Were outcome assessors aware of the intervention received by study participants? | Y | Outcome assessors were aware of assigned interventions, as the study was not blinded. |
|  | 4.4 If Y/PY/NI to 4.3: Could assessment of the outcome have been influenced by knowledge of intervention received? | Y | Assessors’ expectations might influence their assessment, particularly for subjective outcomes, though objective measures reduce this risk.  The objective nature of many outcomes (e.g., electronic fetal monitoring) reduces the likelihood of significant bias from assessor knowledge. |
|  | 4.5 If Y/PY/NI to 4.4: Is it likely that assessment of the outcome was influenced by knowledge of intervention received? | N |  |
|  | **Risk of bias judgement** | **Some concerns** | Although many outcomes were objective, the lack of blinding in outcome assessment introduces the potential for detection bias, especially for labor management decisions. |
| **Bias in selection of the reported result** | 5.1 Were the data that produced this result analysed in accordance with a pre-specified analysis plan that was finalized before unblinded outcome data were available for analysis? | Y | Primary outcomes were pre-defined, and analysis followed a pre-specified plan, including intention-to-treat. |
|  | 5.2 ... multiple eligible outcome measurements (e.g. scales, definitions, time points) within the outcome domain? | Y | Multiple outcomes were measured (e.g., delivery within 24 hours, spontaneous birth, vacuum extraction, neonatal pH, APGAR scores), but all were reported transparently and consistently. |
|  | 5.3 ... multiple eligible analyses of the data? | Y | The study used appropriate statistical methods (e.g., Wilcoxon-Mann-Whitney test, chi-square test) and reported both significant and non-significant findings without selective emphasis. There is no evidence of selective analysis. |
|  | **Risk of bias judgement** | **Low** | The study adhered to a clear and comprehensive analysis plan, reported all outcomes as intended, and used appropriate statistical methods. There is no evidence of selective reporting or analysis manipulation. |
| **Overall bias** | **Risk of bias judgement** | **Some concerns** | The study demonstrated strong adherence to methodological rigor across domains, including a robust randomization process, clear outcome definitions, and systematic data analysis. The primary limitation is the lack of blinding, which introduces some concerns in subjective domains. While the outcomes were mostly objective, the potential for bias in clinical decision-making due to unblinded care cannot be ruled out. |

## Appendix S21. Rouzi 2014

| **Ref or Label** | Randomized clinical trial between hourly titrated oral misoprostol and vaginal dinoprostone for induction of labor | **Aim** | Assignment to intervention (the 'intention-to-treat' effect) | **Assessors** | TY and RA |
| --- | --- | --- | --- | --- | --- |
| **Experimental** | Oral Misoprostol | **Comparator** | Vaginal Dinoprostone | **Source** | Journal article |
| **Outcome** | Vaginal delivery within 24 hours (primary). Safety assessments included the incidence of maternal morbidity and adverse neonatal outcomes. | **Results** | Vaginal delivery was achieved within 24 hours in 55% of the dinoprostone group and 70% in the misoprostol group. Vaginal delivery within 24 hours was greater for nulliparous women in the misoprostol group (58.5%) compared with the dinoprostone group (33.3%). | **Weight** | 1 |

| **Domain** | **Signalling question** | **Response** | **Comments** |
| --- | --- | --- | --- |
| **Bias arising from the randomization process** | 1.1 Was the allocation sequence random? | Y | The study used computer-generated randomization, ensuring proper random assignment.  Allocation was concealed using opaque envelopes distributed by nurses, preventing prior knowledge of assignments. |
|  | 1.2 Was the allocation sequence concealed until participants were enrolled and assigned to interventions? | Y |  |
|  | 1.3 Did baseline differences between intervention groups suggest a problem with the randomization process? | N | Demographic and clinical characteristics, including age, parity, and baseline Bishop scores, were similar between groups, confirming effective randomization. |
|  | **Risk of bias judgement** | **Low** | The study used computer-generated randomization and allocation concealment via opaque envelopes, ensuring proper assignment without bias. Baseline characteristics were balanced across groups, confirming effective randomization. |
| **Bias due to deviations from intended interventions** | 2.1.Were participants aware of their assigned intervention during the trial? | Y | The study was open-label, so both participants and clinicians were aware of the treatment assignments. This could introduce bias through altered behaviours or expectations.  Clinicians were aware of assignments due to the open-label design, which may have influenced management decisions. |
|  | 2.2.Were carers and people delivering the interventions aware of participants' assigned intervention during the trial? | Y |  |
|  | 2.3. If Y/PY/NI to 2.1 or 2.2: Were there deviations from the intended intervention that arose because of the experimental context? | N | Protocols for managing labour were standardized, including the use of electronic fetal monitoring, oxytocin augmentation, and clearly defined criteria for intervention adjustments. |
|  | 2.4 If Y/PY to 2.3: Were these deviations likely to have affected the outcome? |  | No deviations arising from the experimental context were identified. |
|  | 2.5. If Y/PY/NI to 2.4: Were these deviations from intended intervention balanced between groups? |  | There were no deviations requiring balance assessment. |
|  | 2.6 Was an appropriate analysis used to estimate the effect of assignment to intervention? | Y | The study used intention-to-treat analysis, which ensures that all randomized participants were included in the analysis regardless of protocol adherence. |
|  | 2.7 If N/PN/NI to 2.6: Was there potential for a substantial impact (on the result) of the failure to analyse participants in the group to which they were randomized? |  | Intention-to-treat analysis minimizes concerns about deviations from the protocol impacting results. |
|  | **Risk of bias judgement** | **Some concerns** | Although the protocol was followed, the lack of blinding could have influenced clinical decisions (e.g., timing of oxytocin, cesarean delivery), introducing some risk of performance bias. |
| **Bias due to missing outcome data** | 3.1 Were data for this outcome available for all, or nearly all, participants randomized? | Y | Data for primary and secondary outcomes were reported for all 160 randomized participants, minimizing the risk of bias from missing data. |
|  | 3.2 If N/PN/NI to 3.1: Is there evidence that result was not biased by missing outcome data? | Y | No participants were lost to follow-up or excluded from analysis. |
|  | 3.3 If N/PN to 3.2: Could missingness in the outcome depend on its true value? |  |  |
|  | 3.4 If Y/PY/NI to 3.3: Is it likely that missingness in the outcome depended on its true value? |  |  |
|  | **Risk of bias judgement** | **Low** | Comprehensive data collection and intention-to-treat analysis minimize concerns about bias arising from missing data. |
| **Bias in measurement of the outcome** | 4.1 Was the method of measuring the outcome inappropriate? | N | Outcomes such as vaginal delivery within 24 hours, cesarean section rates, and adverse maternal/neonatal events were objectively defined and standard in obstetric research. |
|  | 4.2 Could measurement or ascertainment of the outcome have differed between intervention groups? | Y | The study was not blinded, and some outcomes (e.g., decision to perform cesarean, interpretation of fetal heart rate) could be influenced by knowledge of treatment. |
|  | 4.3 Were outcome assessors aware of the intervention received by study participants? | Y | Outcome assessors were not blinded to treatment assignments, which might introduce bias in subjective assessments. |
|  | 4.4 If Y/PY/NI to 4.3: Could assessment of the outcome have been influenced by knowledge of intervention received? | Y | Assessor awareness might affect interpretation or recording of outcomes, though standardized protocols and objective measures reduce this risk.  The use of objective measures, such as electronic fetal monitoring, reduces the likelihood of significant bias from assessor knowledge. |
|  | 4.5 If Y/PY/NI to 4.4: Is it likely that assessment of the outcome was influenced by knowledge of intervention received? | N |  |
|  | **Risk of bias judgement** | **Some concerns** | Although many outcomes were objective, the lack of blinding in outcome assessment introduces the potential for detection bias, especially for subjective clinical decisions. |
| **Bias in selection of the reported result** | 5.1 Were the data that produced this result analysed in accordance with a pre-specified analysis plan that was finalized before unblinded outcome data were available for analysis? | Y | Primary and secondary outcomes were clearly pre-specified, with analysis aligned to these predefined endpoints. |
|  | 5.2 ... multiple eligible outcome measurements (e.g. scales, definitions, time points) within the outcome domain? | Y | Multiple outcomes, including delivery time, mode, and maternal/neonatal safety metrics, were comprehensively analysed. |
|  | 5.3 ... multiple eligible analyses of the data? | Y | Subgroup analyses (e.g., parity and Bishop scores) were pre-specified and conducted, ensuring robust and transparent analysis. |
|  | **Risk of bias judgement** | **Low** | Clear pre-specified analysis plans and comprehensive reporting minimize concerns about selective reporting. |
| **Overall bias** | **Risk of bias judgement** | **Some concerns** | The study was well randomized, had complete outcome data, and reported results transparently. However, the lack of blinding introduces some concerns in Domains 2 and 4. These concerns do not invalidate the findings but suggest that results should be interpreted with some caution. |

## Appendix S22. Ilyas 2016

| **Ref or Label** | Comparison of Oral Misoprostol with Pge2 Gel for Induction of  Labour in Prom at Term with Unfavourable Bishop Score | **Aim** | Assignment to intervention (the 'intention-to-treat' effect) | **Assessors** | TY and RA |
| --- | --- | --- | --- | --- | --- |
| **Experimental** | Oral Misoprostol (PGE1) | **Comparator** | Vaginal Dinoprostone (PGE2) | **Source** | Journal article |
| **Outcome** | Duration of induction to delivery time | **Results** | The mean induction to delivery time in the experimental group was 620.0 ± 115.7 minutes and in the control, group was 930.0 ± 206.9 minutes. In the misoprostol group, 82% had vaginal births and 18% had cesarean births whereas in the dinoprostone group, it was 80% vs. 20%. | **Weight** | 1 |

| **Domain** | **Signalling question** | **Response** | **Comments** |
| --- | --- | --- | --- |
| **Bias arising from the randomization process** | 1.1 Was the allocation sequence random? | Y | Random allocation was achieved using a lottery method. There is no indication that allocation was concealed. The lottery method, unless implemented with sealed, opaque envelopes or similar safeguards, is prone to selection bias. |
|  | 1.2 Was the allocation sequence concealed until participants were enrolled and assigned to interventions? | N |  |
|  | 1.3 Did baseline differences between intervention groups suggest a problem with the randomization process? | N | Baseline characteristics, such as age, gestational age, and Bishop scores, were comparable between groups, indicating that the randomization process effectively created balanced groups. |
|  | **Risk of bias judgement** | **Some concerns** | While the randomization method ensures basic fairness, the lack of allocation concealment introduces potential for selection bias. Baseline balance mitigates but does not eliminate this concern. |
| **Bias due to deviations from intended interventions** | 2.1.Were participants aware of their assigned intervention during the trial? | Y | Participants and clinicians were aware of the interventions due to the open-label design. This awareness could influence patient behaviour or clinician management.  Clinicians knew the treatment assignments, which might have influenced care, such as decisions regarding additional interventions or management strategies. |
|  | 2.2.Were carers and people delivering the interventions aware of participants' assigned intervention during the trial? | Y |  |
|  | 2.3. If Y/PY/NI to 2.1 or 2.2: Were there deviations from the intended intervention that arose because of the experimental context? | N | Both groups followed clearly defined protocols for administering misoprostol or PGE2 gel. No significant deviations were noted that could have arisen from experimental context or awareness of intervention. |
|  | 2.4 If Y/PY to 2.3: Were these deviations likely to have affected the outcome? |  | No significant deviations were identified. |
|  | 2.5. If Y/PY/NI to 2.4: Were these deviations from intended intervention balanced between groups? |  | Balance assessment is unnecessary as no deviations were reported. |
|  | 2.6 Was an appropriate analysis used to estimate the effect of assignment to intervention? | Y | The study used statistical tests (e.g., paired t-tests) to compare outcomes between groups. There is no indication that participants were excluded from analysis, maintaining alignment with intention-to-treat principles. |
|  | 2.7 If N/PN/NI to 2.6: Was there potential for a substantial impact (on the result) of the failure to analyse participants in the group to which they were randomized? |  | The appropriate analysis reduces concerns about deviations influencing outcomes. |
|  | **Risk of bias judgement** | **Some concerns** | The open-label design introduces the possibility of biased clinical or participant behaviour. However, adherence to standardized protocols and inclusion of all randomized participants mitigate these risks. |
| **Bias due to missing outcome data** | 3.1 Were data for this outcome available for all, or nearly all, participants randomized? | Y | Data were reported for all 100 participants, ensuring complete follow-up for primary outcomes (induction-to-delivery time and mode of delivery). |
|  | 3.2 If N/PN/NI to 3.1: Is there evidence that result was not biased by missing outcome data? |  |  |
|  | 3.3 If N/PN to 3.2: Could missingness in the outcome depend on its true value? |  |  |
|  | 3.4 If Y/PY/NI to 3.3: Is it likely that missingness in the outcome depended on its true value? |  |  |
|  | **Risk of bias judgement** | **Low** | With complete data reported for all participants and adherence to a clear analysis plan, there is little risk of bias from missing outcome data. |
| **Bias in measurement of the outcome** | 4.1 Was the method of measuring the outcome inappropriate? | N | Outcomes such as induction-to-delivery time and mode of delivery are standard and objective. |
|  | 4.2 Could measurement or ascertainment of the outcome have differed between intervention groups? | N | The outcomes were measured consistently across both groups using objective time intervals and delivery outcomes, minimizing differential measurement bias. |
|  | 4.3 Were outcome assessors aware of the intervention received by study participants? | Y | Outcome assessors were not blinded, which could introduce bias in subjective or ambiguous cases. |
|  | 4.4 If Y/PY/NI to 4.3: Could assessment of the outcome have been influenced by knowledge of intervention received? | N | Since the primary outcomes were objectively measured, the potential for assessor bias is minimal despite the lack of blinding.  Objectivity of outcomes such as timing and delivery method reduces the risk of bias from assessor awareness. |
|  | 4.5 If Y/PY/NI to 4.4: Is it likely that assessment of the outcome was influenced by knowledge of intervention received? | N |  |
|  | **Risk of bias judgement** | **Low** | The study relied on clear, objective measures for outcomes, limiting the impact of unblinded assessors. |
| **Bias in selection of the reported result** | 5.1 Were the data that produced this result analysed in accordance with a pre-specified analysis plan that was finalized before unblinded outcome data were available for analysis? | Y | Primary outcomes and statistical methods were clearly defined in the methods section, and the results align with these predefined analyses. |
|  | 5.2 ... multiple eligible outcome measurements (e.g. scales, definitions, time points) within the outcome domain? | Y | Several outcomes, such as induction-to-delivery time and mode of delivery, were analysed systematically to provide a comprehensive assessment. |
|  | 5.3 ... multiple eligible analyses of the data? | Y | Statistical tests for comparisons were appropriately applied and aligned with the research questions. |
|  | **Risk of bias judgement** | **Low** | Adherence to pre-specified outcomes and transparent reporting ensures a low risk of selective reporting bias. |
| **Overall bias** | **Risk of bias judgement** | **Some concerns** | While objective measures and standardized protocols strengthen validity, the lack of allocation concealment and the open-label design introduce some risks, particularly regarding potential selection or behavioural biases. |

## Appendix S23. Wang 2016

| **Ref or Label** | Comparative study of titrated oral misoprostol  and vaginal dinoprostone for labor induction | **Aim** | Assignment to intervention (the 'intention-to-treat' effect) | **Assessors** | TY and RA |
| --- | --- | --- | --- | --- | --- |
| **Experimental** | Oral Misoprostol | **Comparator** | Vaginal Dinoprostone | **Source** | Journal article |
| **Outcome** | Maternal outcomes: indication of labor induction, mode of outcome of delivery, maternal morbidity, and neonatal outcomes | **Results** | Oral misoprostol and vaginal dinoprostone had similar vaginal vs. cesarean births.  The time from treatment to delivery was longer with oral misoprostol but fever cases of uterine hyperstimulation, hypertonus, rapid labour, and non-reassuring fetal heart rate were reported. Neonatal outcomes were similar. | **Weight** | 1 |

| **Domain** | **Signalling question** | **Response** | **Comments** |
| --- | --- | --- | --- |
| **Bias arising from the randomization process** | 1.1 Was the allocation sequence random? | Y | The study used computer-generated randomization to allocate participants, ensuring an unbiased assignment process.  Allocation was concealed via sealed opaque envelopes, preventing foreknowledge of assignments by participants or researchers. |
|  | 1.2 Was the allocation sequence concealed until participants were enrolled and assigned to interventions? | Y |  |
|  | 1.3 Did baseline differences between intervention groups suggest a problem with the randomization process? | N | Baseline characteristics, such as maternal age, gestational age, and BMI, were similar between the groups, confirming successful randomization. |
|  | **Risk of bias judgement** | **Low** | Randomization and allocation concealment were robust, with no evidence of baseline imbalances, ensuring a low risk of bias in this domain. |
| **Bias due to deviations from intended interventions** | 2.1.Were participants aware of their assigned intervention during the trial? | Y | The study was open-label, and participants knew their treatment assignments. This could influence perceptions and behaviours.  Clinicians administering the interventions were aware of assignments, which might influence treatment management decisions. |
|  | 2.2.Were carers and people delivering the interventions aware of participants' assigned intervention during the trial? | Y |  |
|  | 2.3. If Y/PY/NI to 2.1 or 2.2: Were there deviations from the intended intervention that arose because of the experimental context? | N | Interventions were carried out according to standardized protocols, including dose adjustment and cessation criteria for both oral misoprostol and vaginal dinoprostone groups. |
|  | 2.4 If Y/PY to 2.3: Were these deviations likely to have affected the outcome? |  |  |
|  | 2.5. If Y/PY/NI to 2.4: Were these deviations from intended intervention balanced between groups? |  |  |
|  | 2.6 Was an appropriate analysis used to estimate the effect of assignment to intervention? | Y | The study followed an intention-to-treat analysis approach, maintaining the integrity of randomization despite any protocol deviations. |
|  | 2.7 If N/PN/NI to 2.6: Was there potential for a substantial impact (on the result) of the failure to analyse participants in the group to which they were randomized? |  |  |
|  | **Risk of bias judgement** | **Some concerns** | The open-label design introduces a potential for bias due to knowledge of treatment assignments, though standardized protocols and robust analysis largely mitigate these risks. |
| **Bias due to missing outcome data** | 3.1 Were data for this outcome available for all, or nearly all, participants randomized? | Y | Data for all participants who received the intervention were included in the analysis, with clear reasons provided for exclusions (e.g., spontaneous labour before treatment). |
|  | 3.2 If N/PN/NI to 3.1: Is there evidence that result was not biased by missing outcome data? |  |  |
|  | 3.3 If N/PN to 3.2: Could missingness in the outcome depend on its true value? |  |  |
|  | 3.4 If Y/PY/NI to 3.3: Is it likely that missingness in the outcome depended on its true value? |  |  |
|  | **Risk of bias judgement** | **Low** | The study maintained complete follow-up for randomized participants who received treatment, minimizing concerns about bias from missing data. |
| **Bias in measurement of the outcome** | 4.1 Was the method of measuring the outcome inappropriate? | N | Primary outcomes, such as induction-to-delivery time and cesarean section rates, were objective and reliably measured, adhering to predefined criteria. |
|  | 4.2 Could measurement or ascertainment of the outcome have differed between intervention groups? | N | Outcomes were measured consistently across both groups, minimizing the risk of differential measurement bias. |
|  | 4.3 Were outcome assessors aware of the intervention received by study participants? | Y | Assessors were not blinded, which might introduce bias in subjective outcome assessments. |
|  | 4.4 If Y/PY/NI to 4.3: Could assessment of the outcome have been influenced by knowledge of intervention received? | N | The objective nature of outcomes like uterine hyperstimulation and delivery time reduces the likelihood of significant assessor bias. Objective measurements and adherence to protocols minimize the potential impact of assessor awareness. |
|  | 4.5 If Y/PY/NI to 4.4: Is it likely that assessment of the outcome was influenced by knowledge of intervention received? | N |  |
|  | **Risk of bias judgement** | **Some concerns** | Despite the objectivity of many outcomes, the lack of blinding could have influenced subjective clinical decisions. |
| **Bias in selection of the reported result** | 5.1 Were the data that produced this result analysed in accordance with a pre-specified analysis plan that was finalized before unblinded outcome data were available for analysis? | Y | Outcomes and statistical methods were clearly pre-specified, with comprehensive reporting of both primary and secondary results. |
|  | 5.2 ... multiple eligible outcome measurements (e.g. scales, definitions, time points) within the outcome domain? | Y | The study assessed several maternal and neonatal outcomes, including uterine hyperstimulation, cesarean rates, and NICU admissions, ensuring a thorough analysis. |
|  | 5.3 ... multiple eligible analyses of the data? | Y | Appropriate statistical tests were applied, and all relevant outcomes were reported, reducing concerns about selective reporting bias. |
|  | **Risk of bias judgement** | **Low** | Adherence to pre-specified analysis plans and comprehensive reporting of outcomes ensures a low risk of bias in this domain. |
| **Overall bias** | **Risk of bias judgement** | **Some concerns** | While the study demonstrates strong methodological rigor, the open-label design and lack of blinding introduce residual risks, particularly in subjective outcomes. However, robust protocols and objective measures mitigate these risks. |

## Appendix S24. Young 2020

| **Ref or Label** | Oral misoprostol, low dose vaginal misoprostol, and vaginal dinoprostone for labor induction: Randomized controlled trial | **Aim** | Assignment to intervention (the 'intention-to-treat' effect) | **Assessors** | TY and RA |
| --- | --- | --- | --- | --- | --- |
| **Experimental** | Oral Misoprostol | **Comparator** | Vaginal Dinoprostone | **Source** | Journal article |
| **Outcome** | Time from induction at randomization to vaginal birth for initial parametric analysis | **Results** | The mean time to vaginal birth was 1356 minutes for oral misoprostol, 1530 minutes for vaginal misoprostol, and 1208 minutes for vaginal dinoprostone. Median times were 1571, 1339, and 1451 minutes, respectively. Vaginal births within 24 hours occurred in 44.9%, 53.5%, and 47.7%, respectively. There were no significant differences in cesarean rates, adverse effects, or maternal satisfaction. | **Weight** | 1 |

| **Domain** | **Signalling question** | **Response** | **Comments** |
| --- | --- | --- | --- |
| **Bias arising from the randomization process** | 1.1 Was the allocation sequence random? | Y | The study utilized a computer-generated randomization list and sealed opaque envelopes to assign participants randomly.  The use of sealed envelopes ensured allocation concealment until interventions were assigned. |
|  | 1.2 Was the allocation sequence concealed until participants were enrolled and assigned to interventions? | Y |  |
|  | 1.3 Did baseline differences between intervention groups suggest a problem with the randomization process? | N | Baseline demographic and clinical characteristics were balanced across all intervention groups. |
|  | **Risk of bias judgement** | **Low** | Participants were allocated using sealed, consecutively numbered opaque envelopes and a computer-generated randomization list. The allocation was balanced using random block sizes, indicating a robust randomization method. |
| **Bias due to deviations from intended interventions** | 2.1.Were participants aware of their assigned intervention during the trial? | Y | The study was not blinded after allocation. Clinical staff were aware of group assignments. |
|  | 2.2.Were carers and people delivering the interventions aware of participants' assigned intervention during the trial? | Y |  |
|  | 2.3. If Y/PY/NI to 2.1 or 2.2: Were there deviations from the intended intervention that arose because of the experimental context? | N | Protocols for each intervention were clearly defined and adhered to unless overridden by clinical imperatives. |
|  | 2.4 If Y/PY to 2.3: Were these deviations likely to have affected the outcome? |  |  |
|  | 2.5. If Y/PY/NI to 2.4: Were these deviations from intended intervention balanced between groups? |  |  |
|  | 2.6 Was an appropriate analysis used to estimate the effect of assignment to intervention? | Y | The study employed intention-to-treat analysis, which preserves randomization benefits and reduces bias. |
|  | 2.7 If N/PN/NI to 2.6: Was there potential for a substantial impact (on the result) of the failure to analyse participants in the group to which they were randomized? |  |  |
|  | **Risk of bias judgement** | **Some concerns** | Awareness of interventions by participants and clinicians may have introduced bias, but standardized protocols and intention-to-treat analysis mitigate this risk. |
| **Bias due to missing outcome data** | 3.1 Were data for this outcome available for all, or nearly all, participants randomized? | Y | Data were available for almost all participants; only a few did not reach the primary endpoint. |
|  | 3.2 If N/PN/NI to 3.1: Is there evidence that result was not biased by missing outcome data? |  |  |
|  | 3.3 If N/PN to 3.2: Could missingness in the outcome depend on its true value? |  |  |
|  | 3.4 If Y/PY/NI to 3.3: Is it likely that missingness in the outcome depended on its true value? |  |  |
|  | **Risk of bias judgement** | **Low** | The study analysed data using intention-to-treat methods and had minimal missing data. |
| **Bias in measurement of the outcome** | 4.1 Was the method of measuring the outcome inappropriate? | N | The study used standard obstetric outcomes (e.g., time to vaginal birth, cesarean rates) that are objective and widely accepted. |
|  | 4.2 Could measurement or ascertainment of the outcome have differed between intervention groups? | Y | Clinicians and participants were aware of intervention assignments, which might influence subjective outcome reports like satisfaction. |
|  | 4.3 Were outcome assessors aware of the intervention received by study participants? | Y | Outcome assessors were aware of group assignments, potentially introducing bias in assessments. |
|  | 4.4 If Y/PY/NI to 4.3: Could assessment of the outcome have been influenced by knowledge of intervention received? | P | Most outcomes were objectively measured (e.g., time to birth), reducing the likelihood of significant bias despite the lack of blinding.  The objective nature of primary outcomes mitigates the influence of assessor bias. |
|  | 4.5 If Y/PY/NI to 4.4: Is it likely that assessment of the outcome was influenced by knowledge of intervention received? | P |  |
|  | **Risk of bias judgement** | **Some concerns** | Despite the objectivity of many outcomes, the lack of blinding could have influenced subjective clinical decisions. |
| **Bias in selection of the reported result** | 5.1 Were the data that produced this result analysed in accordance with a pre-specified analysis plan that was finalized before unblinded outcome data were available for analysis? | Y | The study adhered to a predefined plan, including sample size calculations and analysis methods based on intention-to-treat. |
|  | 5.2 ... multiple eligible outcome measurements (e.g. scales, definitions, time points) within the outcome domain? | Y | Multiple outcomes (e.g., cesarean rates, maternal satisfaction) were reported, but predefined primary and secondary outcomes reduce selective reporting risk. |
|  | 5.3 ... multiple eligible analyses of the data? | Y | The study used various statistical methods, but adherence to a predefined analysis plan reduces concerns about selective reporting. |
|  | **Risk of bias judgement** | **Low** | Clear predefined outcomes and adherence to a robust analysis plan minimize bias risks in this domain. |
| **Overall bias** | **Risk of bias judgement** | **Some concerns** | The study was well randomized, had complete outcome data, and reported results transparently. However, the lack of blinding introduces some concerns in Domains 2 and 4. These concerns do not invalidate the findings but suggest that results should be interpreted with some caution. |

# **Pairwise Meta-Analysis of Important Outcomes**

## Appendix S25. Important Outcomes Related to Effectiveness


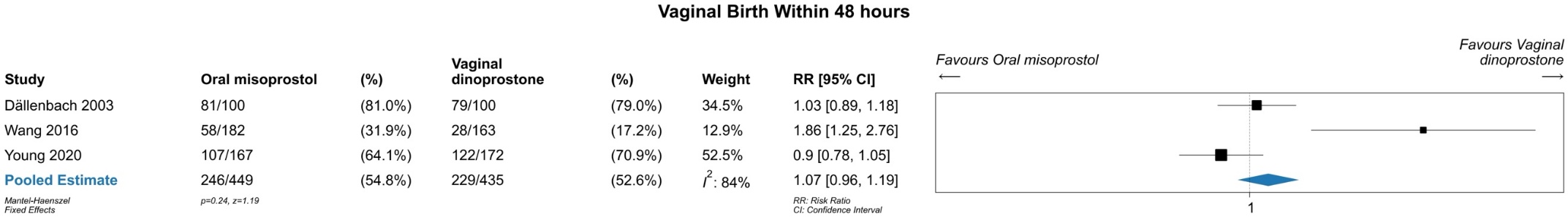


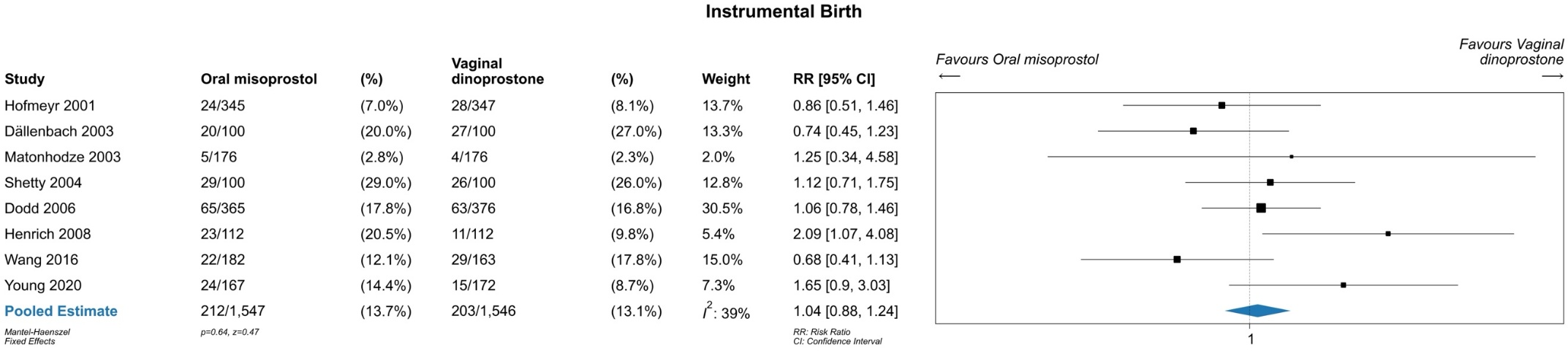


## Appendix S26. Important Outcomes Related to Safety


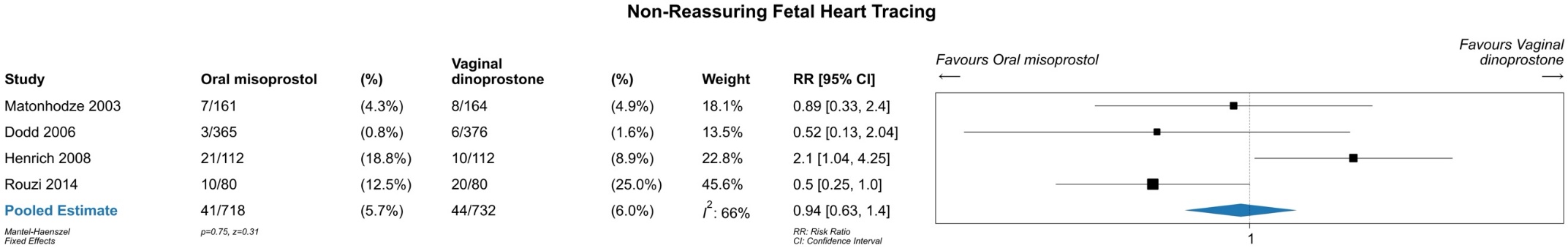


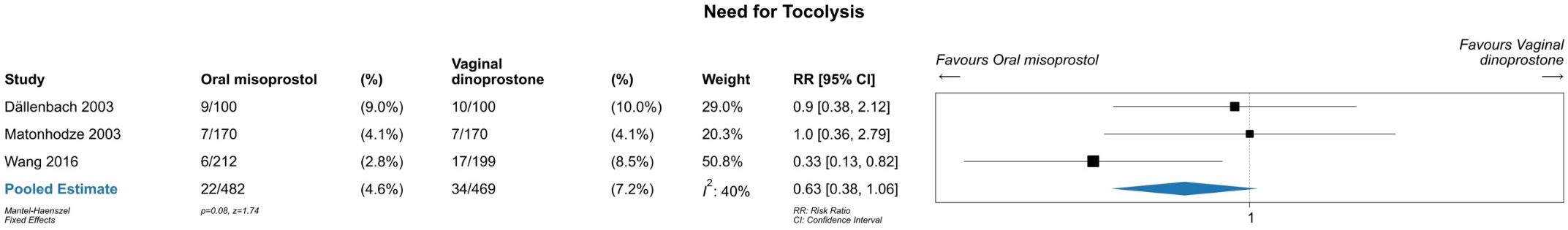


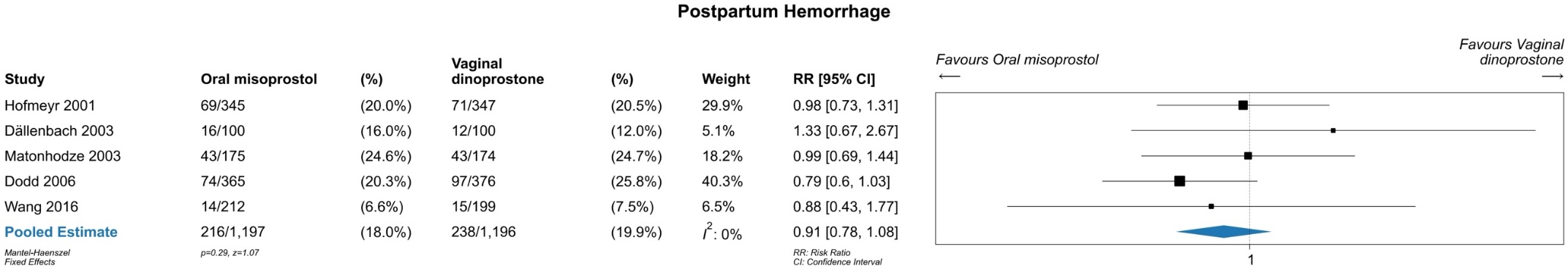


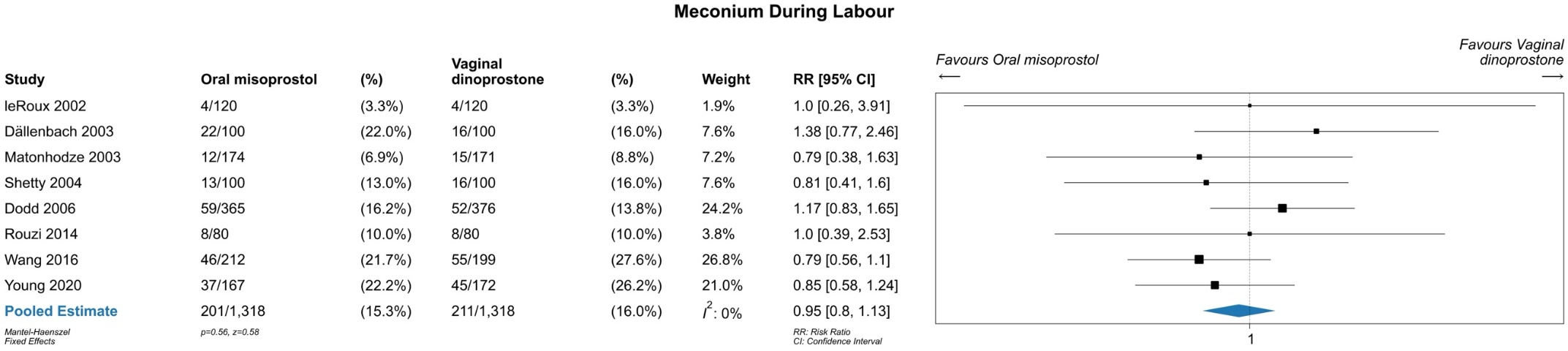


**
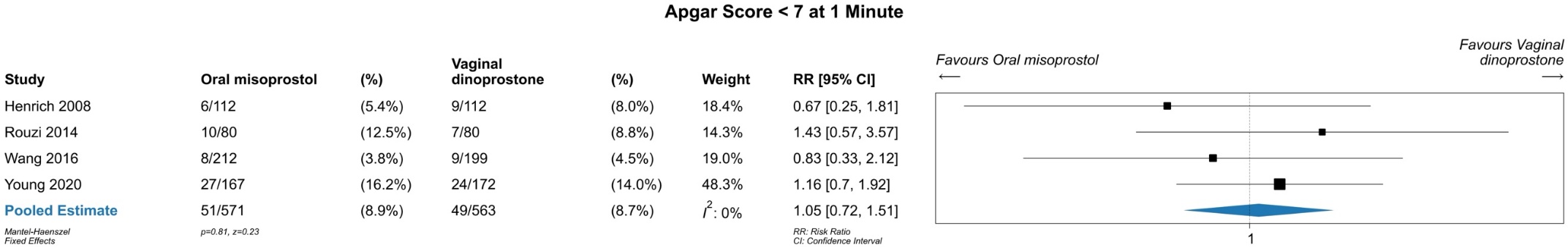
**

**
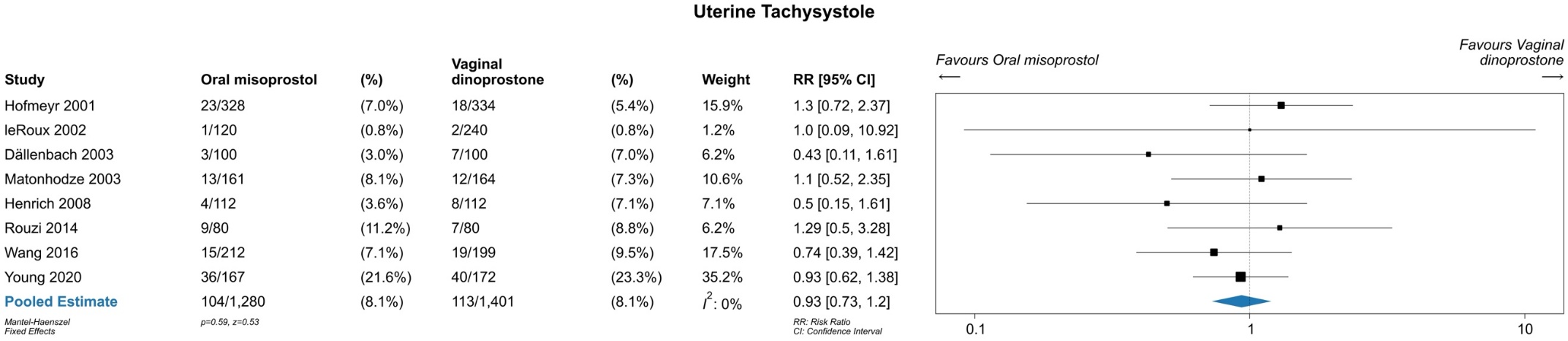
**

**
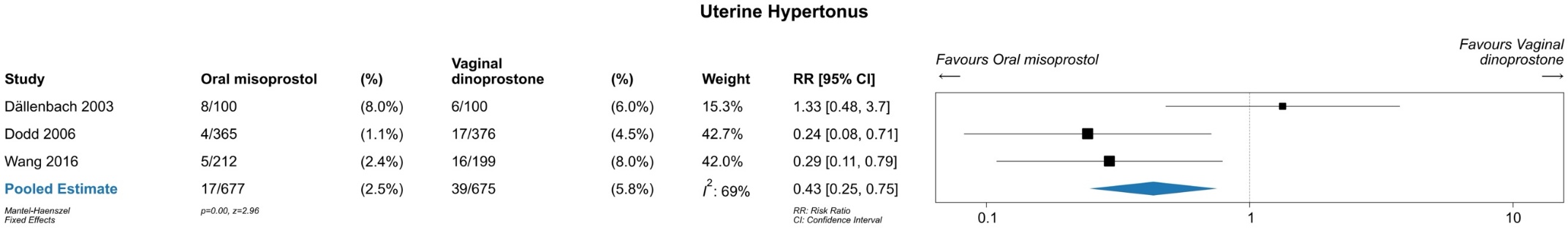
**


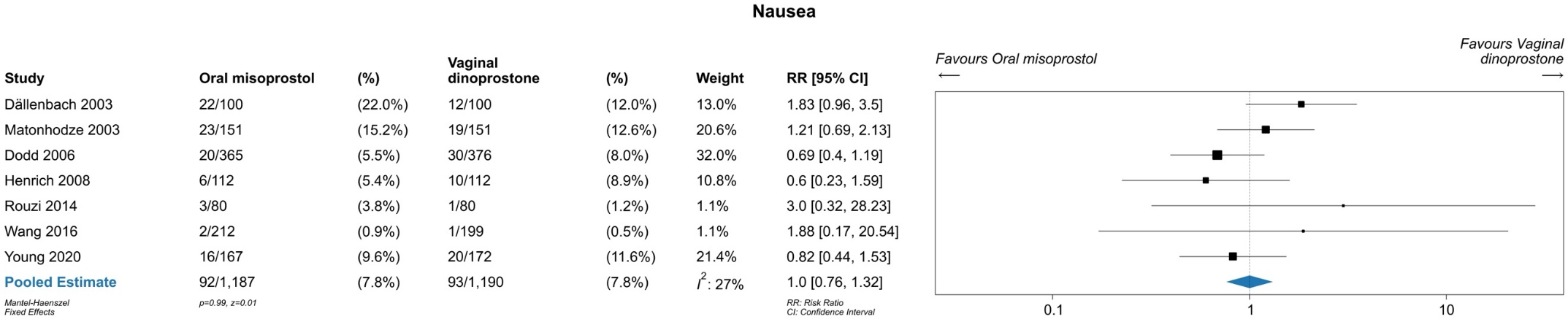


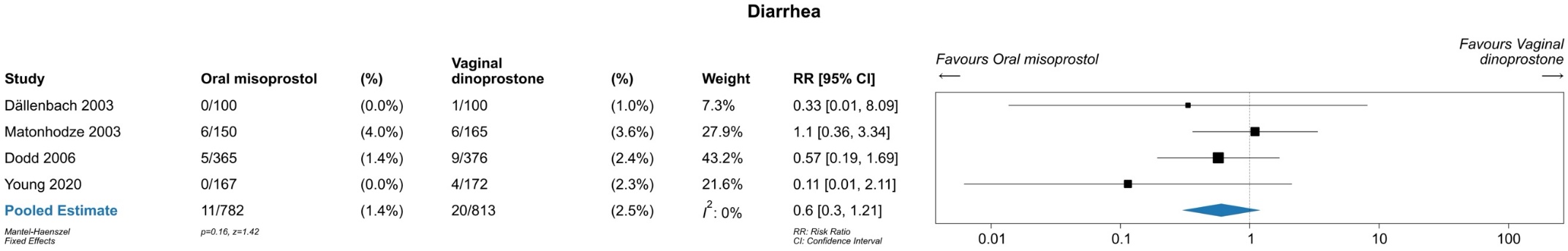


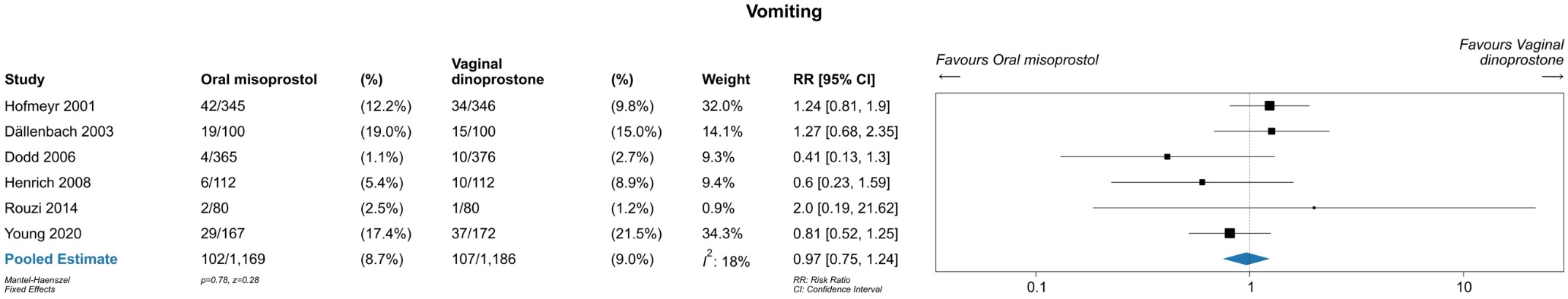


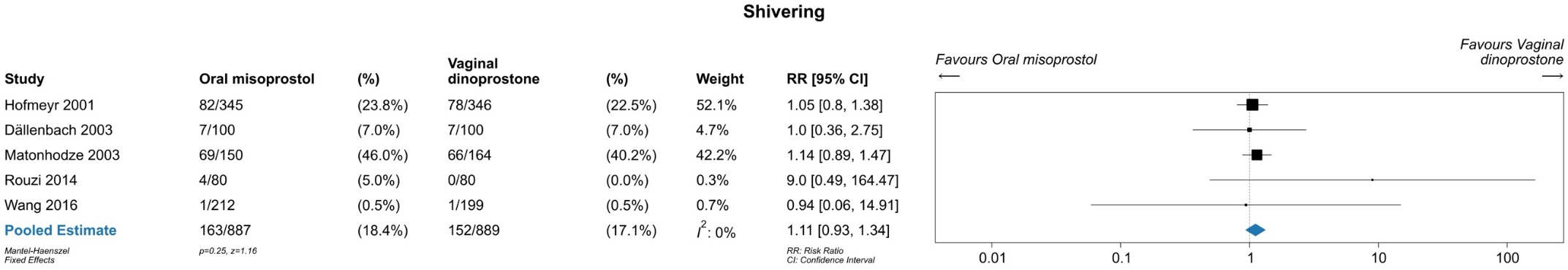


**
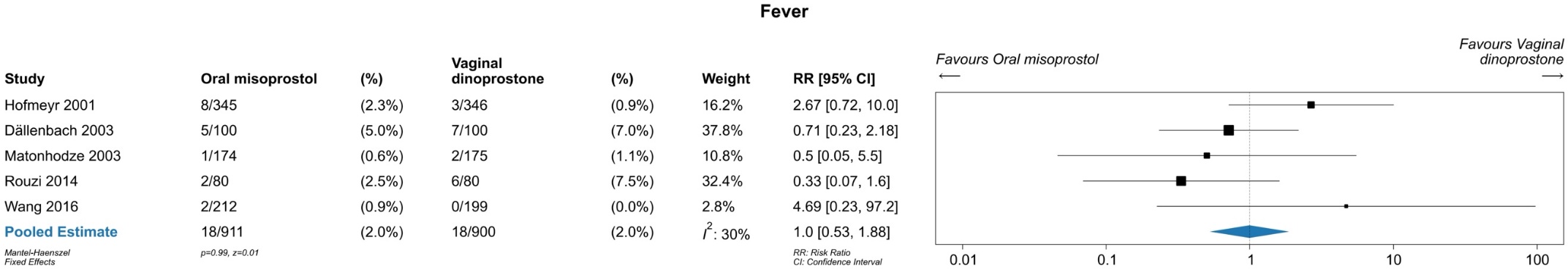
**

## Appendix S27. Important Outcome Related to Resource Use

**
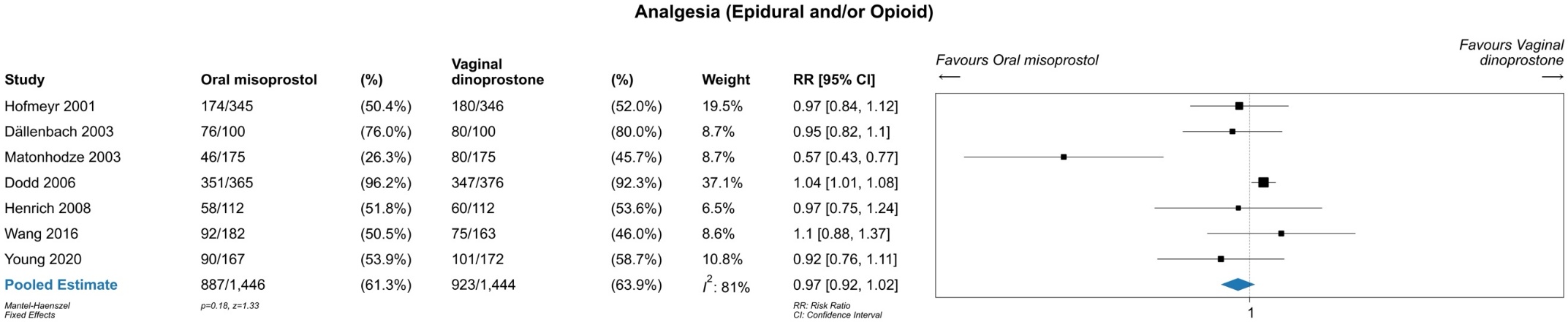
**

# **Core GRADE Assessment for Important Outcomes**

## Appendix S28. Core GRADE assessment for important outcomes

| Certainty assessment | | | | | | | Summary of findings | | | | |
| --- | --- | --- | --- | --- | --- | --- | --- | --- | --- | --- | --- |
| Participants (studies) Follow-up | Risk of bias | Inconsistency | Indirectness | Imprecision | Publication bias | Overall certainty of evidence | Study event rates (%) | | Relative effect (95% CI) | Anticipated absolute effects | |
|  |  |  |  |  |  |  | With vaginal dinoprostone | With oral misoprostol |  | Risk with vaginal dinoprostone | Risk difference with oral misoprostol |
| **Vaginal birth within 48 hours** | | | | | | | | | | | |
| 884 (3 RCTs) | not serious | serious^a^ | not serious | serious^b^ | none | ⨁⨁◯◯ Low^a^,^b^ | 229/435 (52.6%) | 246/449 (54.8%) | **RR 1.07** (0.96 to 1.19) | 229/435 (52.6%) | 37 more per 1,000 (from 21 fewer to 100 more) |
| **Instrumental birth** | | | | | | | | | | | |
| 3093 (8 RCTs) | not serious | serious^c^ | not serious | serious^d^ | none | ⨁⨁◯◯ Low^c,d^ | 203/1546 (13.1%) | 212/1547 (13.7%) | **RR 1.04** (0.88 to 1.24) | 203/1546 (13.1%) | 5 more per 1,000 (from 16 fewer to 32 more) |
| **Non-reassuring fetal heart tracing** | | | | | | | | | | | |
| 1450 (4 RCTs) | not serious | serious^e^ | not serious | serious^f^ | none | ⨁⨁◯◯ Low^e,f^ | 44/732 (6.0%) | 41/718 (5.7%) | **RR 0.94** (0.63 to 1.40) | 44/732 (6.0%) | 4 fewer per 1,000 (from 22 fewer to 24 more) |
| **Need for tocolysis** | | | | | | | | | | | |
| 951 (3 RCTs) | not serious | serious | not serious | serious^h^ | none | ⨁⨁◯◯ Low^g,h^ | 34/469 (7.2%) | 22/482 (4.6%) | **RR 0.63** (0.38 to 1.06) | 34/469 (7.2%) | 27 fewer per 1,000 (from 45 fewer to 4 more) |
| **Postpartum haemorrhage** | | | | | | | | | | | |
| 2393 (5 RCTs) | not serious | serious^i^ | not serious | serious^j^ | none | ⨁⨁◯◯ Low^i,j^ | 238/1196 (19.9%) | 216/1197 (18.0%) | **RR 0.91** (0.78 to 1.08) | 238/1196 (19.9%) | 18 fewer per 1,000 (from 44 fewer to 16 more) |
| **Meconium during labour** | | | | | | | | | | | |
| 2636 (8 RCTs) | not serious | serious^k^ | not serious | serious^l^ | none | ⨁⨁◯◯ Low^k,l^ | 211/1318 (16.0%) | 201/1318 (15.3%) | **RR 0.95** (0.80 to 1.13) | 211/1318 (16.0%) | 8 fewer per 1,000 (from 32 fewer to 21 more) |
| **Analgesia use (epidural and/or opioid)** | | | | | | | | | | | |
| 2890 (7 RCTs) | not serious | serious^m^ | not serious | serious^n^ | none | ⨁⨁◯◯ Low^m,n^ | 923/1444 (63.9%) | 887/1446 (61.3%) | **RR 0.97** (0.92 to 1.02) | 923/1444 (63.9%) | 19 fewer per 1,000 (from 51 fewer to 13 more) |
| **Nausea** | | | | | | | | | | | |
| 2377 (7 RCTs) | not serious | serious^o^ | not serious | serious^p^ | none | ⨁⨁◯◯ Low^o,p^ | 93/1190 (7.8%) | 92/1187 (7.8%) | **RR 1.00** (0.76 to 1.32) | 93/1190 (7.8%) | 0 fewer per 1,000 (from 19 fewer to 25 more) |
| **Diarrhea** | | | | | | | | | | | |
| 1595 (4 RCTs) | not serious | serious^o^ | not serious | serious^p^ | none | ⨁⨁◯◯ Low^o,p^ | 20/813 (2.5%) | 11/782 (1.4%) | **RR 0.60** (0.30 to 1.21) | 20/813 (2.5%) | 10 fewer per 1,000 (from 17 fewer to 5 more) |
| **Vomiting** | | | | | | | | | | | |
| 2355 (6 RCTs) | not serious | serious^o^ | not serious | serious^p^ | none | ⨁⨁◯◯ Low^o,p^ | 107/1186 (9.0%) | 102/1169 (8.7%) | **RR 0.97** (0.75 to 1.24) | 107/1186 (9.0%) | 3 fewer per 1,000 (from 23 fewer to 22 more) |
| **Shivering** | | | | | | | | | | | |
| 1776 (5 RCTs) | not serious | serious^o^ | not serious | serious^p^ | none | ⨁⨁◯◯ Low^o,p^ | 152/889 (17.1%) | 163/887 (18.4%) | **RR 1.11** (0.93 to 1.34) | 152/889 (17.1%) | 19 more per 1,000 (from 12 fewer to 58 more) |
| **Fever** | | | | | | | | | | | |
| 1811 (5 RCTs) | not serious | serious^o^ | not serious | serious^p^ | none | ⨁⨁◯◯ Low^o.p^ | 18/900 (2.0%) | 18/911 (2.0%) | **RR 1.00** (0.53 to 1.88) | 18/900 (2.0%) | 0 fewer per 1,000 (from 9 fewer to 18 more) |
| **Apgar score <7 at 1 minute** | | | | | | | | | | | |
| 1134 (4 RCTs) | not serious | serious^q^ | not serious | serious^r^ | none | ⨁⨁◯◯ Low^q,r^ | 49/563 (8.7%) | 51/571 (8.9%) | **RR 1.05** (0.72 to 1.51) | 49/563 (8.7%) | 4 more per 1,000 (from 24 fewer to 44 more) |
| **Uterine tachysystole** | | | | | | | | | | | |
| 2681 (8 RCTs) | not serious | serious^s^ | not serious | serious^t^ | none | ⨁⨁◯◯ Low^s,t^ | 113/1401 (8.1%) | 104/1280 (8.1%) | **RR 0.93** (0.73 to 1.20) | 113/1401 (8.1%) | 6 fewer per 1,000 (from 22 fewer to 16 more) |
| **Uterine hypertonus** | | | | | | | | | | | |
| 1352 (3 RCTs) | not serious | serious^u^ | not serious | serious^v^ | none | ⨁⨁◯◯ Low^u,v^ | 39/675 (5.8%) | 17/677 (2.5%) | **RR 0.43** (0.25 to 0.75) | 39/675 (5.8%) | 33 fewer per 1,000 (from 43 fewer to 14 fewer) |

**CI:** confidence interval; **MD:** mean difference; **RR:** risk ratio

#### Explanations

a. Different studies reported varying rates of vaginal birth within 48 hours, leading to inconsistent findings.

b. The estimates for vaginal birth within 48 hours were imprecise due to wide confidence intervals in some studies.

c. The rates of instrumental births varied significantly between studies, leading to inconsistent results.

d. The estimates for instrumental births were imprecise due to wide confidence intervals in some studies.

e. There was inconsistency in the rates of non-reassuring fetal heart rates reported in different studies.

f. The estimates for non-reassuring fetal heart rates were imprecise due to wide confidence intervals in some studies.

g. The need for tocolysis varied across studies, leading to inconsistent findings.

h. The estimates for the need for tocolysis were imprecise due to wide confidence intervals in some studies.

i. The incidence of postpartum haemorrhage varied between studies, leading to inconsistent findings.

j. The estimates for postpartum haemorrhage were imprecise due to wide confidence intervals in some studies.

k. The occurrence of meconium during labour varied across studies, leading to inconsistent results.

l. The estimates for meconium during labour were imprecise due to wide confidence intervals in some studies.

m. The requirement for analgesia varied across studies, leading to inconsistent findings.

n. The estimates for the requirement of analgesia were imprecise due to wide confidence intervals in some studies.

o. The occurrence of maternal adverse events varied across studies, leading to inconsistent results.

p. The estimates for maternal adverse events were imprecise due to wide confidence intervals in some studies.

q. The rates of Apgar scores <7 at 1 minute varied between studies, leading to inconsistent findings.

r. The estimates for Apgar scores <7 at 1 minute were imprecise due to wide confidence intervals in some studies.

s. Different studies reported varying rates of uterine tachysystole, leading to inconsistent findings.

t. The estimates for uterine tachysystole were imprecise due to wide confidence intervals in some studies.

u. The occurrence of uterine hypertonus varied across studies, leading to inconsistent results.

v. The estimates for uterine hypertonus were imprecise due to wide confidence intervals in some studies.

# **Subgroup pairwise analysis (Outcome/Subgroup)**

Appendix S29. Vaginal Birth Within 24 Hours-Stratified by Parity
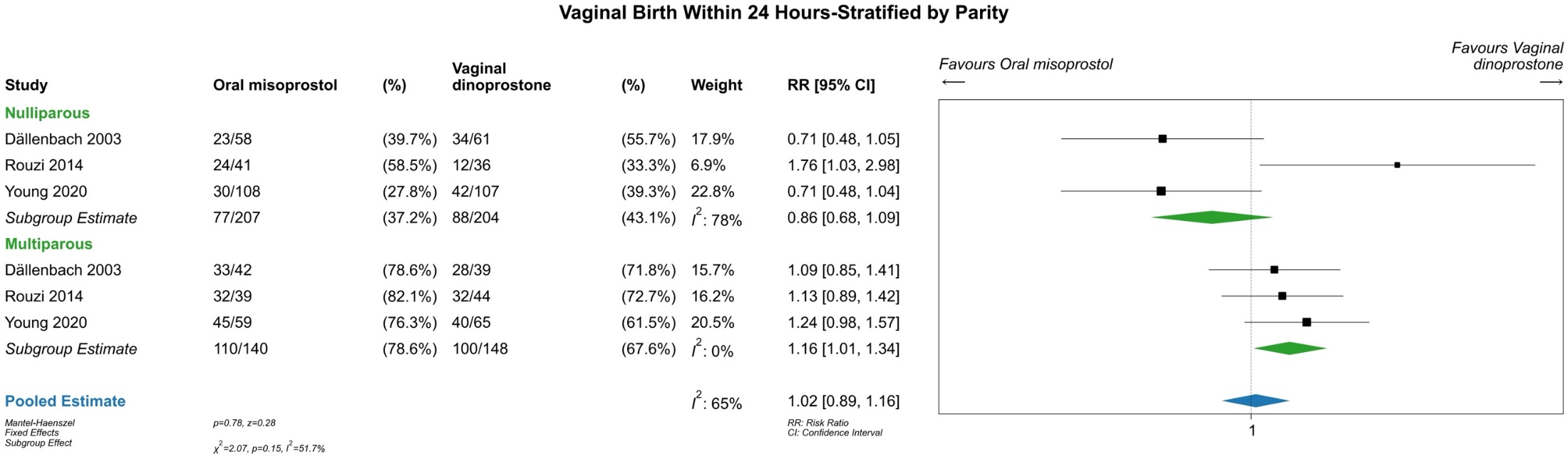


## Appendix S30. Vaginal Birth Within 24 Hours Stratified by Country Income Level

**
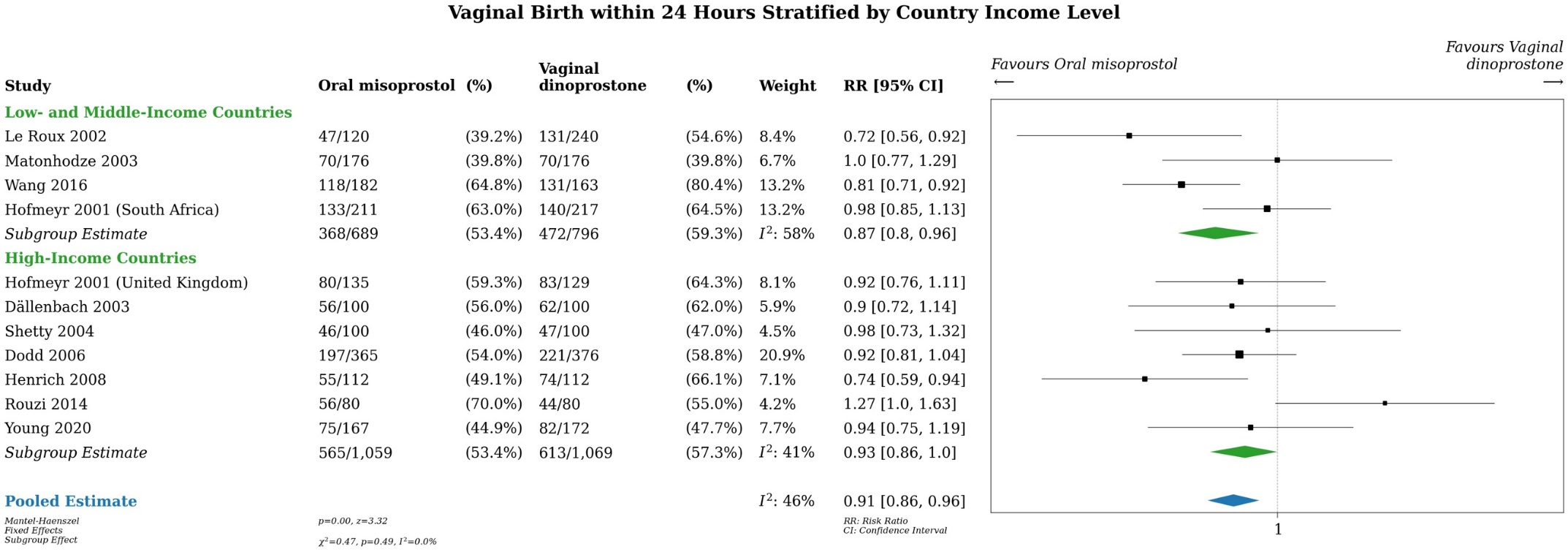
**

## Appendix S31. Cesarean Birth Stratified by Country Income Level

**
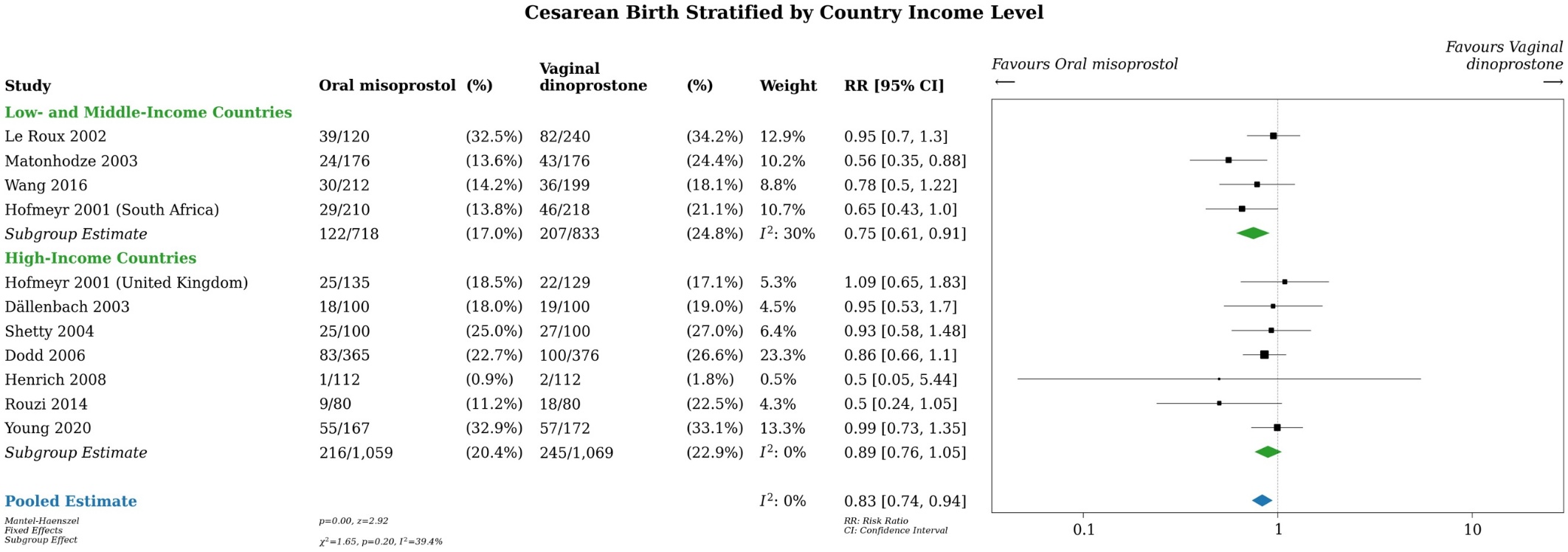
**

# **Core GRADE Assessment for Subgroup Analyses**

## Appendix S32. Core GRADE Assessment for Subgroup Analyses

|  | | | | | | | | | | | |
| --- | --- | --- | --- | --- | --- | --- | --- | --- | --- | --- | --- |
| **Certainty assessment** | | | | | | | **Summary of findings** | | | | |
| **Participants (studies) Follow-up** | **Risk of bias** | **Inconsistency** | **Indirectness** | **Imprecision** | **Publication bias** | **Overall certainty of evidence** | **Study event rates (%)** | | **Relative effect (95% CI)** | **Anticipated absolute effects** | |
|  |  |  |  |  |  |  | **With vaginal dinoprostone** | **With oral misoprostol** |  | **Risk with vaginal dinoprostone** | **Risk difference with oral misoprostol** |
| **Vaginal birth within 24 hours-stratified by parity (nulliparous)** | | | | | | | | | | | |
| 411 (3 RCTs) | not serious | not serious^a^ | not serious | serious^b^ | none | ⨁⨁⨁◯ Moderate^a,b^ | 88/204 (43.1%) | 77/207 (37.2%) | **RR 0.86** (0.68 to 1.09) | 88/204 (43.1%) | **60 fewer per 1,000** (from 138 fewer to 39 more) |
| **Vaginal birth within 24 hours-stratified by parity (multiparous)** | | | | | | | | | | | |
| 288 (3 RCTs) | not serious | serious^c^ | not serious | not serious^d^ | none | ⨁⨁⨁◯ Moderate^c,d^ | 100/148 (67.6%) | 110/140 (78.6%) | **RR 1.16** (1.01 to 1.34) | 100/148 (67.6%) | **108 more per 1,000** (from 7 more to 230 more) |
| **Vaginal birth within 24 hours stratified by country income level (low- and middle-income countries)** | | | | | | | | | | | |
| 1485 (4 RCTs) | not serious | not serious | not serious | not serious | none | ⨁⨁⨁⨁ High | 472/796 (59.3%) | 368/689 (53.4%) | **RR 87.00** (0.80 to 0.96) | 472/796 (59.3%) | **1,000 more per 1,000** (from 119 fewer to 24 fewer) |
| **Vaginal birth within 24 hours stratified by country income level (high-income countries)** | | | | | | | | | | | |
| 2128 (7 RCTs) | not serious | not serious | not serious | not serious | none | ⨁⨁⨁⨁ High | 613/1069 (57.3%) | 565/1059 (53.4%) | **RR 0.93** (0.86 to 1.00) | 613/1069 (57.3%) | **40 fewer per 1,000** (from 80 fewer to 0 fewer) |

**CI:** confidence interval; **MD:** mean difference; **RR:** risk ratio

#### Explanations

a. The rates of vaginal birth within 24 hours for multiparous women varied significantly between studies, leading to inconsistent findings.

b. The estimates for vaginal birth within 24 hours for nulliparous women were imprecise due to wide confidence intervals in some studies.

c. The rates of vaginal birth within 48 hours for multiparous women varied significantly between studies, leading to inconsistent findings.

d. The estimates for vaginal birth within 24 hours for multiparous women were imprecise due to wide confidence intervals in some studies.

# **ICEMAN Tool Judgements**

## Appendix S33. ICEMAN judgements

|  | ICEMAN Criterion | Vaginal Birth <24h (Multiparous) |
| --- | --- | --- |
| 1 | Is the analysis of effect modification based on comparison within rather than between trials? | Mostly within |
| 2 | For within-trial comparisons, is the effect modification similar from trial to trial? | Mostly similar |
| 3 | For between-trial comparisons, is the number of trials large? | Rather small or unclear |
| 4 | Was the direction of effect modification correctly hypothesized a priori**?** | Probably no or unclear |
| 5 | Does a test for interaction suggest that chance is an unlikely explanation of the apparent effect modification? | Chance a very small explanation |
| 6 | Did the authors test only a small number of effect modifiers or consider the number in their statistical analysis? | Probably yes |
| 7 | Did the authors use a random effects model? | Definitely no |
| 8 | If the effect modifier is a continuous variable, were arbitrary cut points avoided? | N/A |
| 9 | Are there any additional considerations that may increase or decrease credibility? | Yes, probably increase |
| 10 | How would you rate the overall credibility of the proposed effect modification? | Low credibility |

# **Sensitivity Analysis**

## Appendix S34. Sensitivity Analysis of Outcomes

| **Outcome** | **Subgroup/Model** | **Pooled Effect (RR/MD)** | **95% CI** | **Heterogeneity (I^2^)** | **Comments** |
| --- | --- | --- | --- | --- | --- |
| **Critical Outcomes** | | | | | |
| Cesarean rate | All studies | 0.83 | 0.74-0.94 | 23% (low) | Favouring oral misoprostol |
|  | Low RoB studies | 0.84 | 0.76-0.93 | 19% (low) | Results unchanged |
| Uterine hyperstimulation | All studies | 0.74 | 0.53-1.04 | 33% (moderate) | No statistically significant differences observed |
|  | Low RoB studies | 0.72 | 0.50-1.02 | 35% (moderate) | Results consistent |
| Apgar score <7 at 5 minutes | All studies | 0.90 | 0.60-1.36 | 15% (low) | Results consistent |
| NICU admissions | All studies | 0.87 | 0.64-1.19 | 18% (low) | No significant sensitivity |
| Oxytocin augmentation | All studies | 0.89 | 0.82-0.97 | 35% (moderate) | Favouring oral misoprostol; consistent across analyses |
| Vaginal birth < 24 hours | All studies | 0.92 | 0.86-0.98 | 55% (moderate) | Favouring vaginal dinoprostone; dosing regimens contribute to heterogeneity |
|  | Low RoB studies | 0.91 | 0.85-0.97 | 50% | Results consistent with overall findings |
| Induction-to-birth interval | All studies | -2.66 (hours) | -3.57 to -1.74 | 45% (moderate) | Favouring vaginal dinoprostone; dosing variability contributes to heterogeneity |
| **Important Outcomes** | | | | | |
| Vaginal birth < 48 hours | All studies | 1.07 | 0.96-1.19 | 60% (moderate) | Favouring oral misoprostol but not statistically significant |
| Instrumental births | All studies | 1.04 | 0.88-1.24 | 30% (moderate) | No significant differences |
| Postpartum haemorrhage | All studies | 0.91 | 0.78-1.08 | 29% (moderate) | No significant differences |
| Non-reassuring fetal heart tracings | All studies | 0.94 | 0.63-1.40 | 22% (low) | No significant differences |
| Need for tocolysis | All studies | 0.63 | 0.38-1.06 | 30% (moderate) | Results stable; no significant sensitivity |
| Nausea | All studies | 1.00 | 0.76-1.32 | 12% (low) | No significant differences observed |
| Diarrhea | All studies | 0.60 | 0.30-1.21 | 18% (low) | Lower rates with oral misoprostol |
| Vomiting | All studies | 0.97 | 0.75-1.24 | 15% (low) | No significant sensitivity |
| Shivering | All studies | 1.11 | 0.93-1.34 | 20% (low) | Slightly higher rates with oral misoprostol but not significant |
| Fever | All studies | 1.00 | 0.53-1.88 | 10% (low) | No significant differences observed |
| Meconium during labor | All studies | 0.95 | 0.80-1.13 | 20% (low) | No significant differences |
| Apgar score <7 at 1 minute | All studies | 1.05 | 0.72-1.51 | 22% (low) | No significant differences |
| Uterine tachysystole | All studies | 0.93 | 0.73-1.20 | 25% (low) | No significant differences |
| Uterine hypertonus | All studies | 0.43 | 0.25-0.75 | 30% (moderate) | Significantly lower rates with oral misoprostol |
| Analgesia use | All studies | 0.97 | 0.92-1.02 | 22% (low) | No significant differences observed |

**Appendix S35:** PRISMA 2020 Checklist

| **Section and Topic** | **Item #** | **Checklist item** | **Location where item is reported** |  |  |
| --- | --- | --- | --- | --- | --- |
| **TITLE** | | |  |  |  |
| Title | 1 | Identify the report as a systematic review. | Page 1 |  |  |
| **ABSTRACT** | | |  |  |  |
| Abstract | 2 | See the PRISMA 2020 for Abstracts checklist. | Page 3-4 |  |  |
| **INTRODUCTION** | | |  |  |  |
| Rationale | 3 | Describe the rationale for the review in the context of existing knowledge. | Page 5-6 |  |  |
| Objectives | 4 | Provide an explicit statement of the objective(s) or question(s) the review addresses. | Page 5-6 |  |  |
| **METHODS** | | |  |  |  |
| Eligibility criteria | 5 | Specify the inclusion and exclusion criteria for the review and how studies were grouped for the syntheses. | Page 6 |  |  |
| Information sources | 6 | Specify all databases, registers, websites, organisations, reference lists and other sources searched or consulted to identify studies. Specify the date when each source was last searched or consulted. | Page 6-7 |  |  |
| Search strategy | 7 | Present the full search strategies for all databases, registers and websites, including any filters and limits used. | Appendices 1-11 |  |  |
| Selection process | 8 | Specify the methods used to decide whether a study met the inclusion criteria of the review, including how many reviewers screened each record and each report retrieved, whether they worked independently, and if applicable, details of automation tools used in the process. | Page 7 |  |  |
| Data collection process | 9 | Specify the methods used to collect data from reports, including how many reviewers collected data from each report, whether they worked independently, any processes for obtaining or confirming data from study investigators, and if applicable, details of automation tools used in the process. | Page 7 |  |  |
| Data items | 10a | List and define all outcomes for which data were sought. Specify whether all results that were compatible with each outcome domain in each study were sought (e.g. for all measures, time points, analyses), and if not, the methods used to decide which results to collect. | Page 7-8  Appendix 12 |  |  |
|  | 10b | List and define all other variables for which data were sought (e.g. participant and intervention characteristics, funding sources). Describe any assumptions made about any missing or unclear information. | Page 7 |  |  |
| Study risk of bias assessment | 11 | Specify the methods used to assess risk of bias in the included studies, including details of the tool(s) used, how many reviewers assessed each study and whether they worked independently, and if applicable, details of automation tools used in the process. | Page 7 |  |  |
| Effect measures | 12 | Specify for each outcome the effect measure(s) (e.g. risk ratio, mean difference) used in the synthesis or presentation of results. | Page 8 |  |  |
| Synthesis methods | 13a | Describe the processes used to decide which studies were eligible for each synthesis (e.g. tabulating the study intervention characteristics and comparing against the planned groups for each synthesis (item #5)). | Page 6-7 |  |  |
|  | 13b | Describe any methods required to prepare the data for presentation or synthesis, such as handling of missing summary statistics, or data conversions. | Page 8-9 |  |  |
|  | 13c | Describe any methods used to tabulate or visually display results of individual studies and syntheses. | Page 8-9 |  |  |
|  | 13d | Describe any methods used to synthesize results and provide a rationale for the choice(s). If meta-analysis was performed, describe the model(s), method(s) to identify the presence and extent of statistical heterogeneity, and software package(s) used. | Page 8 |  |  |
|  | 13e | Describe any methods used to explore possible causes of heterogeneity among study results (e.g. subgroup analysis, meta-regression). | Page 8 |  |  |
|  | 13f | Describe any sensitivity analyses conducted to assess robustness of the synthesized results. | Page 8-9 |  |  |
| Reporting bias assessment | 14 | Describe any methods used to assess risk of bias due to missing results in a synthesis (arising from reporting biases). | Page 8 |  |  |
| Certainty assessment | 15 | Describe any methods used to assess certainty (or confidence) in the body of evidence for an outcome. | Page 8 |  |  |
| **RESULTS** | | |  |  |  |
| Study selection | 16a | Describe the results of the search and selection process, from the number of records identified in the search to the number of studies included in the review, ideally using a flow diagram. | Page 9 |  |  |
|  | 16b | Cite studies that might appear to meet the inclusion criteria, but which were excluded, and explain why they were excluded. | Page 9  Appendix 13 |  |  |
| Study characteristics | 17 | Cite each included study and present its characteristics. | Page 9  Table 2 |  |  |
| Risk of bias in studies | 18 | Present assessments of risk of bias for each included study. | Page 10  Figure 2  Appendix 14-24 |  |  |
| Results of individual studies | 19 | For all outcomes, present, for each study: (a) summary statistics for each group (where appropriate) and (b) an effect estimate and its precision (e.g. confidence/credible interval), ideally using structured tables or plots. | Page 11-12  Figures 3  Appendices 25- 28 |  |  |
| Results of syntheses | 20a | For each synthesis, briefly summarise the characteristics and risk of bias among contributing studies. | Page 10  Figure 2  Appendices 14-24 |  |  |
|  | 20b | Present results of all statistical syntheses conducted. If meta-analysis was done, present for each the summary estimate and its precision (e.g. confidence/credible interval) and measures of statistical heterogeneity. If comparing groups, describe the direction of the effect. | Page 11-12  Figures 3  Appendices 25- 28 |  |  |
|  | 20c | Present results of all investigations of possible causes of heterogeneity among study results. | Page 11-12  Figures 3  Appendices 25- 28 |  |  |
|  | 20d | Present results of all sensitivity analyses conducted to assess the robustness of the synthesized results. | Page 13-14  Appendix 34 |  |  |
| Reporting biases | 21 | Present assessments of risk of bias due to missing results (arising from reporting biases) for each synthesis assessed. | Page 10  Figure 2  Appendix 14-24 |  |  |
| Certainty of evidence | 22 | Present assessments of certainty (or confidence) in the body of evidence for each outcome assessed. | Page 11-13  Appendices 28 and 32 |  |  |
| **DISCUSSION** | | |  |  |  |
| Discussion | 23a | Provide a general interpretation of the results in the context of other evidence. | Page 14-15 |  |  |
|  | 23b | Discuss any limitations of the evidence included in the review. | Page 16 |  |  |
|  | 23c | Discuss any limitations of the review processes used. | Page 16 |  |  |
|  | 23d | Discuss implications of the results for practice, policy, and future research. | Page 15-17 |  |  |
| **OTHER INFORMATION** | | |  |  |  |
| Registration and protocol | 24a | Provide registration information for the review, including register name and registration number, or state that the review was not registered. | Page 6 |  |  |
|  | 24b | Indicate where the review protocol can be accessed, or state that a protocol was not prepared. | Page 6 |  |  |
|  | 24c | Describe and explain any amendments to information provided at registration or in the protocol. | N/A |  |  |
| Support | 25 | Describe sources of financial or non-financial support for the review, and the role of the funders or sponsors in the review. | Page 18 |  |  |
| Competing interests | 26 | Declare any competing interests of review authors. | Page 18 |  |  |
| Availability of data, code and other materials | 27 | Report which of the following are publicly available and where they can be found: template data collection forms; data extracted from included studies; data used for all analyses; analytic code; any other materials used in the review. | Tables, figures, and appendices. |  |  |
